# Blood biomarkers indicate that the preclinical stages of Alzheimer's disease present overlapping molecular features

**DOI:** 10.1038/s41598-020-71832-y

**Published:** 2020-09-24

**Authors:** Alfonso Di Costanzo, Debora Paris, Dominique Melck, Antonella Angiolillo, Gaetano Corso, Mauro Maniscalco, Andrea Motta

**Affiliations:** 1 1Centre for Research and Training in Medicine for Aging, Department of Medicine and Health Sciences “Vincenzo Tiberio”, University of Molise, 86100 Campobasso, Italy; 2 Institute of Biomolecular Chemistry, National Research Council, 80078 Pozzuoli, Naples Italy; 3 Department of Clinical and Experimental Medicine, University of Foggia, 71122 Foggia, Italy; 4 Pulmonary Rehabilitation Unit, ICS Maugeri SpA SB, Institute of Telese Terme, 82037 Telese Terme, Benevento Italy

**Keywords:** Predictive markers, Molecular medicine

## Abstract

It is still debated whether non-specific preclinical symptoms of Alzheimer’s disease (AD) can have diagnostic relevance. We followed the evolution from cognitively normal to AD by NMR-based metabolomics of blood sera. Multivariate statistical analysis of the NMR profiles yielded models that discriminated subjective memory decline (SMD), mild cognitive impairment (MCI) and AD. We validated a panel of six statistically significant metabolites that predicted SMD, MCI and AD in a blind cohort with sensitivity values ranging from 88 to 95% and receiver operating characteristic values from 0.88 to 0.99. However, lower values of specificity, accuracy and precision were observed for the models involving SMD and MCI, which is in line with the pathological heterogeneity indicated by clinical data. This excludes a “linear” molecular evolution of the pathology, pointing to the presence of overlapping “gray-zones” due to the reciprocal interference of the intermediate stages. Yet, the clear difference observed in the metabolic pathways of each model suggests that pathway dysregulations could be investigated for diagnostic purposes.

## Introduction

Alzheimer’s disease (AD) represents the principal neuronal dysfunction. In 2015, *ca.* 50 million people lived with AD worldwide, to reach 75 million by 2030 and progress to 132 million by 2050, causing a dramatic increase of the annual healthcare costs (https://www.alz.co.uk/research/WorldAlzheimerReport2015.pdf). The administration of drugs to symptomatic AD patients shows no clinical benefits, most likely because the treatments start too late throughout the pathological process^1^. In addition, the beginning of the pharmacological treatment is also delayed because “older adults are inadequately assessed for cognitive impairment during routine visits with their primary care providers”^2^.

The AD “continuum” from cognitively normal (CN) subjects, begins with a Subjective Memory Decline (SMD), and via Mild Cognitive Impairment (MCI) reaches AD^3^, with SMD timed 5–11 years, and MCI detected 1 to 5 years before reaching dementia. MCI subjects may not evolve into dementia as part of them revert to CN or do not progress to MCI, which is usually considered the first stage of dementia, including AD. An open question is if subtle cognitive changes produce “molecular signs” before clinical manifestations appear. Current state-of-the-art diagnostic tools analyze invasively biomatrices like cerebrospinal fluid (CSF), are costly like brain imaging, challenging (neuropsychological screening questionnaire) and often of restricted availability, while functional diagnostics requires noninvasivity and cost-effective tools to map the evolution of cognitive disorders.

Since about half a liter per day of CSF is drained from the brain into the blood, and such a molecular leakage is certainly helped by the damaged blood–brain barrier of AD^4^, blood can be considered a valuable biomatrix to investigate brain neurodegeneration^5,6^. Support to this comes from a comparison between CSF and plasma, which identified common metabolic pathways for MCI and AD in both fluids^7^. Recently, the development of MCI/AD in older adults was predicted by using plasma phospholipids^8^, but the use of the same technical platform and the same markers failed to replicate the results^9^.

The above results confirm that “there is a signal in the blood: but the question remains, can that signal be translated to a replicable and useful biomarkers?”^10^. In addition, blood accumulates markers from all organs and tissues, and the presence of comorbidities and age-related medications certainly interfere^11^. Presently, no robust and trustable blood-based biomarkers are available for diagnostic purposes. This opens several questions: Is this related to clinical heterogeneity of AD? Are biomarkers representative of a specific phenotype? Which is the acceptable prediction limit of a model based on blood biomarkers for the AD progression and diagnosis?

In this paper, nuclear magnetic resonance (NMR)-based metabolomics of sera from CN, SMD, MCI and AD subjects was used to verify the presence of preclinical markers characterizing the progression to AD. Metabolomics investigates the disease molecular mechanisms and can distinguish phenotypical differences. Metabolites are downward products of transcriptome and proteome, and therefore they represent a more specific framework to understand complex biological outcomes^12^. We obtained specific statistical models that in a blind external cohort of subjects predicted groups of patients with sensitivity values ranging from 88 to 95% and receiver operating characteristic (ROC) values from 0.88 to 0.99. However, lower values of specificity, accuracy and precision were obtained for the models involving SMD and MCI. This is in line with the pathological heterogeneity indicated by clinical data, and rules out a “linear” molecular evolution of the pathology, pointing to the presence of overlapping “gray-zones” between intermediate stages.

## Results

### Demographic characteristics of the study subjects

**Figure 1:**
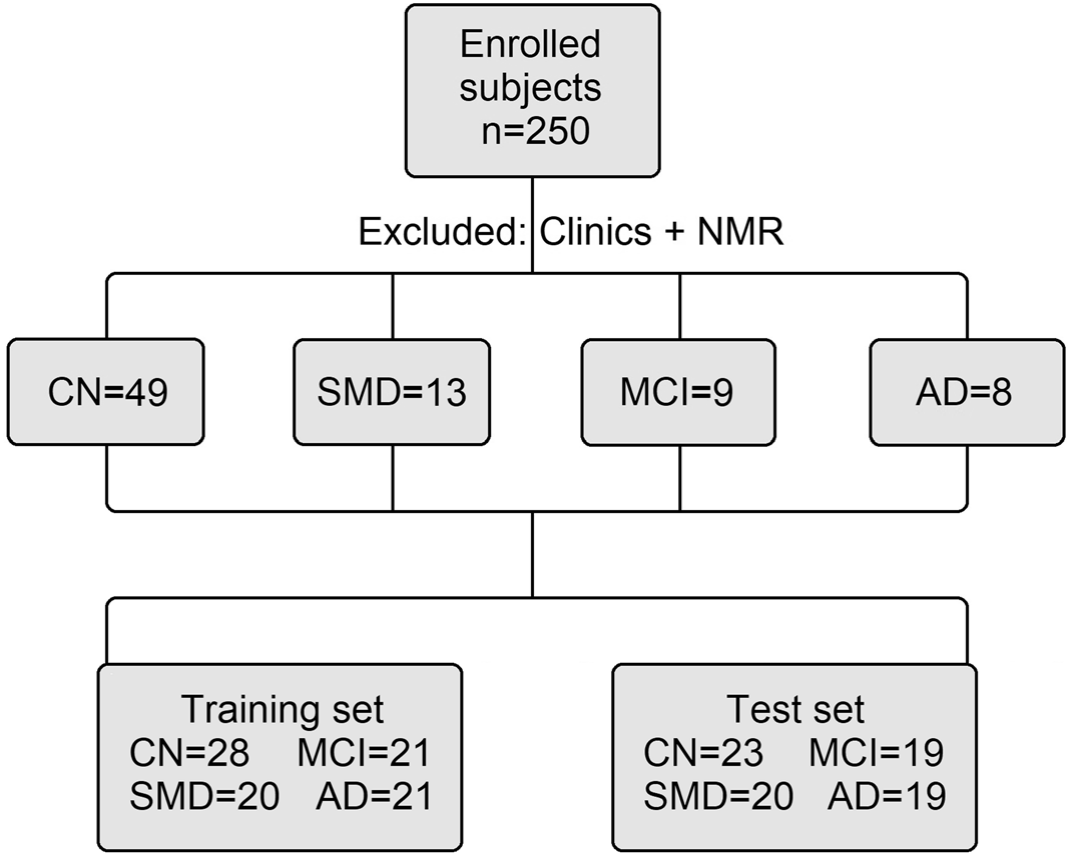
Flow diagram illustrating the study design. For clinical and NMR exclusion creiteria see text. CN, cognitively normal subjects; SMD, subjective memory decline; MCI, mild cognitive impairment; and AD, Alzheimer’s disease.

We consecutively recruited 250 study participants. After evaluation of the inclusion/exclusion criteria and acquisition of NMR spectra of serum samples (see Methods), we excluded 49 CN, 13 SMD, 9 MCI and 8 AD samples. The schematic diagram illustrating the overall study design is reported in Fig. 1.

Selected subjects were randomly allocated in two groups: the first (the training group) comprised 90 patients and was used to generate the statistical models based on NMR data; the second (the test group, not considered for the primary analysis) included 81 patients, and was used as a control set to verify blindly the models’ reliability. The training set included 28 CN (15 F, 13 M), 20 SMD (13 F, 7 M), 21 MCI (13 F, 8 M), and 21 AD (14 F, 7 M). The same four classes were present in the test set, which comprised 23 CN (10 F, 13 M), 20 SMD (13 F, 7 M), 19 MCI (13 F, 6 M), and 19 AD (13 F, 6 M) (Fig. 1). The patient distribution was unknown to the NMR and statistical analysis people.

Subjects’ characteristics and results of dedicated statistical analysis are provided in Table 1 for the training set and in Table 2 for the test set. The effects of comorbidities and pharmacological treatments were not statistically significant. Major exceptions were age, education level, BMI and MMSE data (Tables 1 and 2). Accordingly, we applied ANCOVA (Analysis of Covariance) to determine the impact on the discriminating metabolomic biomarkers of statistically significant covariates (see below).

**Table 1:** Characteristics of the subjects enrolled in the training set^a^.

TRAINING SET	1: CN	2: SMD	3: MCI	4: AD	*p* value 1–2; 1–3; 1–4	*p* value 2–3; 2–4; 3–4
**Anthropometric data**						
No	28	20	21	21	–	–
Age (mean ± SD; y)	63.5 ± 5.3	65.1 ± 7.7	68.2 ± 7.1	79.1 ± 6.4	0.39; **0.01**; < **0.001**	0.18; < **0.001**; < **0.001**
Sex (F; %/M; %)	14; 50.0/14; 50.0	14; 70.0/6; 30.0	16; 76.2/5; 23.8	15; 71.4/6; 28.6	0.63; 0.48; 0.48	0.8; > 0.99; > 0.99
Education level (mean ± SD; y)	13.1 ± 3.0	11.9 ± 3.6	9.5 ± 5.3	9.4 ± 6.1	0.21; **0.004**; **0.007**	0.12; 0.11; 0.6
BMI (mean ± SD; kg/m^2^)	28.3 ± 3.9	26.9 ± 5.1	27.7 ± 3.0	23.1 ± 4.1	0.28; 0.56; < **0.001**	0.5; **0.01**; < **0.001**
MMSE (mean ± SD; score)	29.0 ± 1.8	29.2 ± 1.7	25.5 ± 3.7	*15.2* ± *6.8*	0.69; < **0.001**; < **0.001**	** < 0.001**; < **0.001**; < **0.001**
Familial AD (n; %)	3; 10.7	3; 15.0	2; 9.5	*2; 9.5*	> 0.99; > 0.99; > 0.99	> 0.99; > 0.99; > 0.99
Familial Dementia (n; %)	2; 7.1	4; 20.0	6; 28.6	*5; 23.8*	0,38; 0.13; 0.2	0.73; > 0.99; 0.8
Familial Neurodegenerative Diseases (n; %)	2; 7.1	4; 20.0	3; 14.3	*5; 23.8*	0.38; 0.53; 0.2	0.7; > 0.99; 0.7
Smoke^b^ (n; %)	11; 39.3	8; 40.0	8; 38.1	*4; 19.0*	> 0.99; > 0.99; 0.36	> 0.99; 0.4; 0.39
Dyslipidemia (n; %)	10; 35.7	7; 35.0	7; 33.3	5; 23.8	> 0.99; > 0.99; 0.5	> 0.99; 0.5; 0.52
Diabetes (n; %)	6; 21.4	3; 15.0	3; 14.3	2; 9.5	0.72; 0.72; 0.45	> 0.99; > 0.99; > 0.99
Hypertension (n; %)	10; 35.7	10; 50.0	10; 47.6	5; 23.8	0.59; 0.57; 0.56	> 0.99; 0.36; 0.36
Arrhythmia (n; %)	4; 14.3	5; 20.0	2; 9.5	0; 0.0	0.48; > 0.99; 0.14	0.41; 0.053; 0.48
AED (n; %)	0; 0.0	0; 0.0	1; 4.8	4; 19.0	> 0.99; 0.44; 0.04	> 0.99; 0.11; 0.35
Myocardial Infarction (n; %)	1; 3.6	1; 5.0	2; 9.5	1; 4.8	> 0.99; > 0.99; > 0.99	> 0.99; > 0.99; > 0.99
Heart Failure^c^ (n; %)	2; 7.1	1; 5.0	2; 9.5	1; 4.8	> 0.99; > 0.99; > 0.99	> 0.99; > 0.99; > 0.99
TIA/Stroke (n; %)	0; 0.0	0; 0.0	2; 9.5	1; 4.8	> 0.99; 0.44; 0.19	> 0.99; > 0.99; > 0.99
Chronic Kidney Disease^d^ (n; %)	0; 0.0	1; 5.0	0; 0.0	1; 4.8	> 0.99; > 0.99; > 0.99	> 0.99; > 0.99; > 0.99
Prior Tumors (n; %)	4; 14.3	3; 15.0	2; 9.5	3; 14.3	> 0.99; > 0.99; > 0.99	> 0.99; > 0.99; > 0.99
Neurodegenerative Diseases (n; %)	0; 0.0	0; 0.0	1; 4.8	0; 0.0	> 0.99; > 0.99; > 0.99	> 0.99; > 0.99; > 0.99
Alcool > 4 unit/day (n; %)	1; 3.6	0; 0.0	0; 0.0	0; 0.0	> 0.99; > 0.99; > 0.99	> 0.99; > 0.99; > 0.99
Carotid Atheroma or Aneurysms (n; %)	3; 10.7	2; 10.0	2; 9.5	4; 19.0	> 0.99; > 0.99; > 0.99	> 0.99; > 0.99; > 0.99
Venous Insufficiency (n; %)	3; 10.7	3; 15.0	1; 4.8	3; 14.3	> 0.99; 0.63; > 0.99	0.6; > 0.99; 0.6
Asthma, COPD, O_2_ Therapy (n; %)	0; 0.0	2; 10.0	1; 4.8	2; 9.5	0.18; 0.44; 0.19	> 0.99; > 0.99; > 0.99
Dysthyroidism (n; %)	9; 32.1	5; 25.0	3; 14.3	1; 4.8	0.76; 0.33; 0.07	0.7; 0.19; 0.60
Cirrhosis, Steatosis, Biliary Lithiasis (n; %)	3; 10.7	3; 15.0	1; 4.8	2; 9.5	> 0.99; 0.63; > 0.99	0.6; > 0.99 > 0.99
Previous Surgery (n; %)	10; 35.7	10; 50.0	4; 19.0	3; 14.3	0.59; 0.37; 0.33	0.21; 0.11; > 0.99
**Pharmacological Treatment**						
Antihypertensive (n; %)	10; 35.7	9; 45.0	10; 47.6	5; 23.8	0.78; 0.60; 0.56	> 0.99; 0.36; 0.37
Lipid-lowering (n; %)	6; 21.4	5; 25.0	6; 28.6	2; 9.5	> 0.99; 0.75; 0.45	> 0.99; 0.41; 0.36
Hypoglycemic (n; %)	3; 10.7	2; 10.0	2; 9.5	1; 4.8	> 0.99; > 0.99; 0.63	> 0.99; 0.5; 0.5
Antiplatelet (n; %)	4; 15.7	3; 15.0	2; 9.5	5; 23.8	> 0.99; > 0.99; 0.71	> 0.99; > 0.99; 0.42
Thyroid Hormones (n; %)	6; 21.4	4; 20.0	2; 9.5	1; 4.8	0.99; 0.45; 0.22	0.66; 0.34; > 0.99
Antianxiety (n; %)	2; 7.1	0; 0.0	0; 0.0	4; 19.0	0.78; 0.75; 0.39	> 0.99; 0.17; 0.17
Antidepressant (n; %)	2; 7.1	2; 7.1	2; 9.5	7; 33.3	> 0.99; > 0.99; 0.07	0.97; 0.26; 0.3
Neurotrophic (n; %)	1; 3.6	1; 5.0	1; 4.8	4; 19.0	> 0.99; > 0.99; 0.17	> 0.99; 0.3; 0.3
I-AChE (n; %)	0; 0.0	0; 0.0	1; 4.8	2; 9.5	> 0.99; 0.44; 0.19	0.4; 0.7; > 0.99

Bold values are statistically significant (*p* < 0.05).

^a^CN, cognitively normal; SMD, subjective memory decline; MCI, mild cognitive impairment; AD, Alzheimer’s disease; BMI, Body mass index; MMSE, Mini Mental State Examination; AED, antiepileptic drugs; TIA, Transient Ischemic Attack; I-AChE, Acetylcholinesterase Inhibitors.

^b^Current or former smokers.

^c^Subjects in NYHA (New York Heart Association) classes I–II.

^d^Subjects with glomerular filtration rate (GFR) > 30 ml/min/1.73 m^2^.

**Table 2:** Characteristics of the subjects enrolled in the test set^a^.

TEST SET	1: CN	2: SMD	3: MCI	4: AD	*p* value 1–2; 1–3; 1–4	*p* value 2–3; 2–4; 3–4
**Anthropometric data**						
No	23	20	19	19	-	-
Age (mean ± SD; y)	62.8 ± 6.4	64.2 ± 7.3	68.3 ± 5.4	77.5 ± 7.4	0.5; **0.005**; < **0.001**	0.06; < **0.001**; < **0.001**
Sex (F; %/M; %)	11; 47.8/12; 52.2	12; 60.0/8; 40.0	10; 52.6/9; 47.4	12; 63.2/7; 36.8	> 0.99; > 0.99; > 0.99	> 0.99; > 0.99; > 0.99
Education level (mean ± SD; y)	12.9 ± 2.6	12.0 ± 2.8	9.5 ± 3.9	9.0 ± 4.8	0.28; **0.001**; **0.001**	** < 0.001**; < **0.001**; < **0.001**
BMI (mean ± SD; kg/m^2^)	28.8 ± 3.	27.0 ± 3.2	26.9 ± 3.9	24.4 ± 4.4	0.08; 0.09; **0.007**	0.9; **0.04**; 0.07
MMSE (mean ± SD; score)	29.4 ± 2.7	29.2 ± 1.8	24.9 ± 5.5	*15.4* ± *7.1*	0.78; **0.001**; < **0.001**	** < 0.001**; < **0.001**; < **0.001**
Familial AD (n; %)	2; 8.7	2; 10.0	2; 10.5	*2; 10.5*	> 0.99; > 0.99; > 0.99	> 0.99; > 0.99; > 0.99
Familial Dementia (n; %)	1; 4.3	3; 15.0	4; 21.0	*3; 15.8*	0.34;0.18; 0.33	> 0.99; > 0.99; > 0.99
Familial Neurodegenerative Diseases (n; %)	1; 4.3	3; 15.0	2; 10.5	*3; 15.8*	0.34; 0.68; 0.33	> 0.99; > 0.99; > 0.99
Smoke^b^ (n; %)	8; 34.8	8; 40.0	5; 26.3	*2; 10.5*	0.9; 0.75; 0.52	0.74; 0.15; 0.42
Dyslipidemia (n; %)	8; 34.8	6; 30.0	5; 26.3	4; 21.0	> 0.99; 0.75; 0.52	> 0.99; 0.73; > 0.99
Diabetes (n; %)	4; 17.4	2; 10.0	2; 10.5	2; 10.5	0.67; 0.66; 0.67	> 0.99; > 0.99; > 0.99
Hypertension (n; %)	8; 34.8	7; 35.0	8; 42.1	4; 21.0	> 0.99; 0.99; 0.52	> 0.99; 0.51; 0.42
Arrhythmia (n; %)	2; 8.7	5; 25.0	2; 10.5	0; 0.0	0.41; > 0.99; 0.5	0.4; 0.06; 0.48
AED (n; %)	0; 0.0	0; 0.0	0; 0.0	4; 21.0	> 0.99; > 0.99; 0.1	> 0.99; 0.3; 0.2
Myocardial Infarction (n; %)	1; 4.3	1; 5.0	1; 5.3	1; 5.3	> 0.99; > 0.99; > 0.99	> 0.99; > 0.99; > 0.99
Heart Failure^c^ (n; %)	1; 4.3	1; 5.0	1; 5.3	1; 5.3	> 0.99; > 0.99; > 0.99	> 0.99; > 0.99; > 0.99
TIA/Stroke (n; %)	0; 0.0	1; 5.0	2; 10.5	0; 0.0	> 0.99; > 0.99; > 0.99	> 0.99; > 0.99; > 0.99
Chronic Kidney Disease^d^ (n; %)	0; 0.0	1; 5.0	0; 0.0	1; 5.3	> 0.99; > 0.99; > 0.99	> 0.99; > 0.99; > 0.99
Prior Tumors (n; %)	3; 13.0	3; 15.0	1; 5.3	2; 10.5	> 0.99; > 0.99; > 0.99	> 0.99; > 0.99; > 0.99
Neurodegenerative Diseases (n; %)	0; 0.0	0; 0.0	1; 5.3	0; 0.0	> 0.99; > 0.99; > 0.99	> 0.99; > 0.99; > 0.99
Alcool > 4 unit/day (n; %)	0; 0.0	0; 0.0	0; 0.0	0; 0.0	> 0.99; > 0.99; > 0.99	> 0.99; > 0.99; > 0.99
Carotid Atheroma or Aneurysms (n; %)	2; 8.7	1; 5.0	1; 5.3	2; 10.5	> 0.99; > 0.99; > 0.99	> 0.99; > 0.99; > 0.99
Venous Insufficiency (n; %)	2; 8.7	2; 10.0	0; 0.0	2; 10.5	> 0.99; 0.49; > 0.99	> 0.99; 0.49; 0.5
Asthma, COPD, O_2_ herapy (n; %)	1; 4.3	1; 5.0	1; 5.3	1; 5.3	> 0.99; > 0.99; > 0.99	> 0.99; > 0.99; > 0.99
Dysthyroidism (n; %)	8; 34.8	4; 20.0	2; 10.5	1; 5.3	0.51; 0.17; 0.07	0.50; 0.30; 0.80
Cirrhosis, Steatosis, Biliary Lithiasis (n; %)	3; 13.0	2; 10.0	0; 0.0	1; 5.3	> 0.99; 0.25; 0.33	0.49; > 0.99; > 0.99
Previous Surgery (n; %)	11; 47.8	8; 40.0	3; 15.8	3; 15.8	0.78; 0.2; 0.2	0.3; 0.3; > 0.99
**Pharmacological Treatment**						
Antihypertensive (n; %)	9; 39.1	9; 45.0	8; 42.1	6; 31.6	> 0.99; > 0.99; 0.77	0.76; 0.76; 0.75
Lipid-lowering (n; %)	6; 26.1	5; 25.0	5; 26.3	2; 10.5	> 0.99; > 0.99; 0.44	0.99; 0.42; 0.42
Hypoglycemic (n; %)	3; 13.0	2; 10.0	1; 5.3	1; 5.3	> 0.99; 0.44; 0.44	0.51; 0.51; > 0.99
Antiplatelet (n; %)	4; 17.4	3; 15.0	1; 5.3	4; 21.0	> 0.99; 0.37; > 0.99	> 0.99; > 0.99; 0.35
Thyroid Hormones (n; %)	5; 21.7	4; 20.0	1; 5.3	0; 0.0	> 0.99; 0.37; 0.07	0.11; 0.1; > 0.99
Antianxiety (n; %)	1; 4.3	1; 5.0	2; 10.5	4; 21.0	0.36; 0.68; > 0.99	> 0.99; 0.34; 0.66
Antidepressant (n; %)	2; 8.7	1; 5.0	2; 10.5	6; 31.6	> 0.99; > 0.99; 0.24	> 0.99; 0.1; 0.25
Neurotrophic (n; %)	0; 0.0	0; 0.0	1; 5.3	3; 15.8	> 0.99; 0.46; 0.1	> 0.99; 0.23; 0.6
I-AChE (n; %)	0; 0.0	0; 0.0	0; 0.0	2; 10.5	> 0.99; 0.8; 0.7	> 0.99; 0.48; 0.4

Bold values are statistically significant (*p* < 0.05).

^a^CN, cognitively normal; SMD, subjective memory decline; MCI, mild cognitive impairment; AD, Alzheimer’s disease; BMI, Body mass index; MMSE, Mini Mental State Examination; AED, antiepileptic drugs; TIA, Transient Ischemic Attack; I-AChE, Acetylcholinesterase Inhibitors.

^b^Current or former smokers.

^c^Subjects in NYHA (New York Heart Association) classes I–II.

^d^Subjects with glomerular filtration rate (GFR) > 30 ml/min/1.73 m^2^.

**Figure 2:**
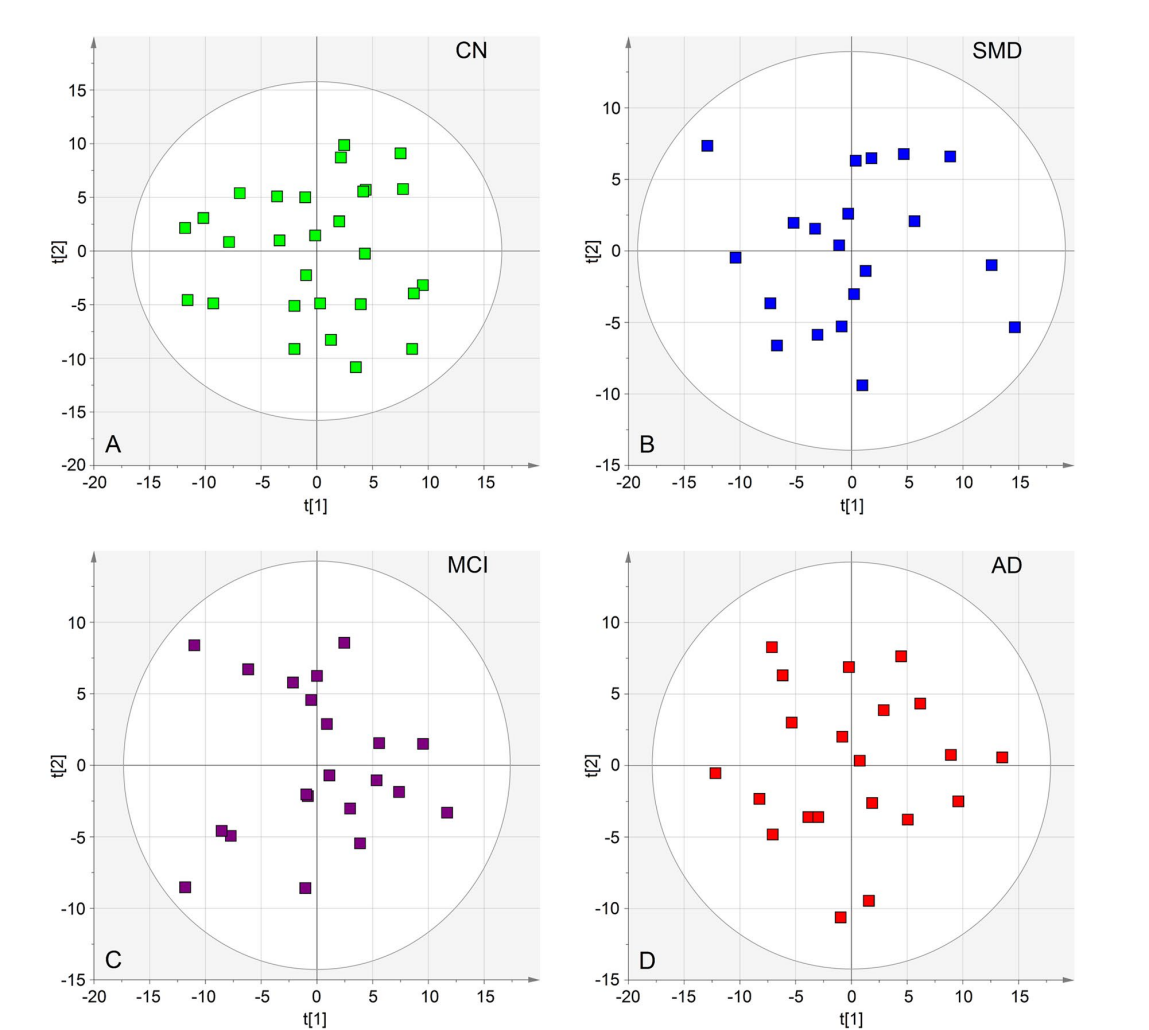
PCA scores plots representing each single class model for the training set (90 total samples). CN, cognitively normal subjects (**A**, green squares); SMD, subjective memory decline (**B**, blue squares); MCI, mild cognitive impairment (**C**, purple squares); and AD, Alzheimer’s disease (red squares). All class-models indicate that the samples are homogeneous as no outliers were detected. The labels t[1] and t[2] along the axes represent the scores (the first 2 partial least-squares components) of the model, which are sufficient to build a satisfactory classification model.

During the study period, there were no changes in medication or comorbidity exacerbation in any of the subjects. However, to check for possible interference, we first applied principal component analysis (PCA), which is an unsupervised method that requires no prior knowledge of the data set, to a subset of each group of samples (28 CN controls, 20 SMD subjects, 21 MCI and 21 AD) in order to detect possible outliers and/or subgroups. Two-component PCA models (Fig. 2) were generated for all classes, obtaining the following quality parameters: CN, R^2^ = 0.410; Q^2^ = 0.240; SMD, R^2^ = 0.410; Q^2^ = 0.174; MCI, R^2^ = 0.372; Q^2^ = 0.122; and AD, R^2^ = 0.384; Q^2^ = 0.184 (Table S1). Cumulative R^2^ and Q2 represent the goodness-of-fit and the goodness-of-prediction parameters, measuring how well the model fits the data, and how well the model predicts new data, respectively. For R^2^ and Q^2^ acceptable values must be ≥ 0.5, with |R^2^—Q^2^|< 0.2–0.3. In each model, no discernible patterns were identified, neither subgroups nor strong outliers. Thus, none of the variables (presence of comorbidity, therapeutic treatment, age, sex, etc.) generated interference. Therefore, all of the 90 samples were included in the training set. The PCA analysis was also applied to all 171 (training and validation) samples, and no discernible patterns and/or outliers were detected (Fig. S1).

### Class separation in the AD continuum

Figure S2 shows the NMR profiles (spectra) of serum samples from CN (Fig. S2A), SMD (Fig. S2B), MCI (Fig. S2C) and AD (Fig. S2D) subjects. For each sample, we acquired two types of one-dimensional (1D) proton spectra, namely, a standard one, and a Carr–Purcell–Meiboom–Gill (CPMG). The latter was used to simplify the spectra by relying on the difference between transverse T_2_ relaxation time of macromolecules and metabolites and therefore avoid standard chemical separation methods. These spectra were used for multivariate analysis. The signals (resonances) were assigned to single metabolites by resorting to 2D NMR experiments (not shown), sample spiking with corresponding chemical standards, and literature data. Table S2 reports the assignments of the identified metabolites.

Orthogonal projections to latent structures discriminant analysis (OPLS-DA) was then applied to investigate a two-group classification at time (Table S3). Only the CN–AD (R^2^ = 0.747; Q^2^ = 0.428; *p* = 0.0004), CN–MCI (R^2^ = 0.422; Q^2^ = 0.053; *p* = 0.039), SMD-AD (R^2^ = 0.617; Q^2^ = 0.355; *p* = 0.002), and MCI-AD (R^2^ = 0.594; Q^2^ = 0.178; *p* = 0.01) models presented statistical significance. The others, including the all-class (CN–SMD–MCI–AD, R^2^ = 0.376; Q^2^ = 0.094; *p* = 0.90), were not considered as the relative OPLS regressions generated unreliable models (Table S3). The models showed a good class separation along the predictive component (*x*-axis), especially for the CN–AD and SMD–AD comparisons (bi-correlation plots in Fig. 2A,C, respectively), while some MCI samples overlap with CN (Fig. 2B) and AD (Fig. 2D) groups. Graphical overlap reflects the “metabolic overlap” related to the MCI status, which does not always evolve into dementia (see below).

**Figure 3:**
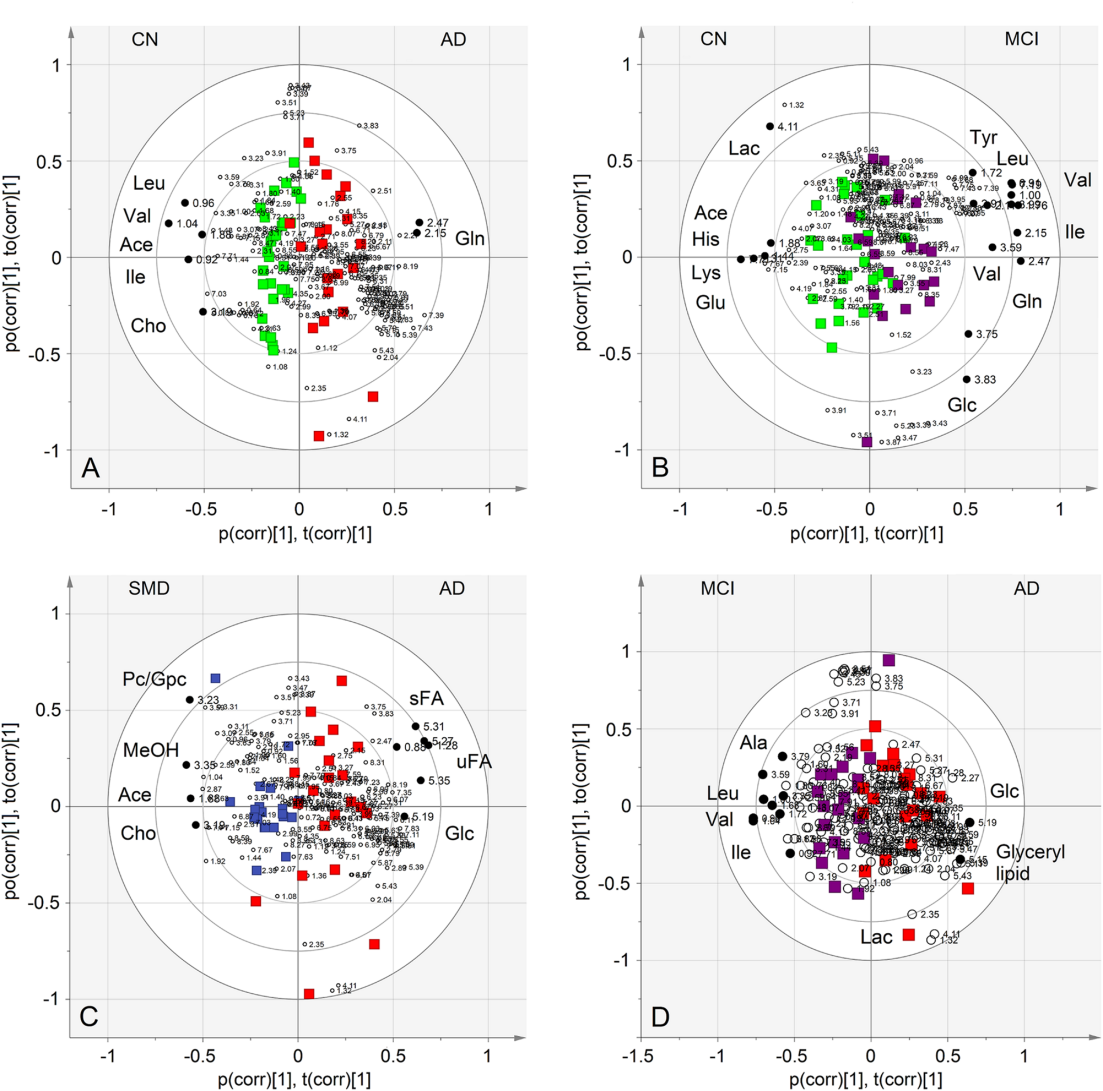
Biplot representing the co-chart of loadings with samples and covariance for each single class model of the training set: (**A**), CN subjects (green squares) *vs.* AD patients (red squares); (**B**), CN (green squares) *vs.* MCI subjects (purple squares); (**C**), MCI (purple squares) *vs.* AD (red squares); (**D**), SMD subjects (blue squares) *vs.* AD (red squares). For each model, relevant signals were highlighted (black dots, VIP > 1 and |*p*corr|≥ 0.6). Ace, acetate; Leu, leucine; Val, valine; Ile, isoleucine; Gln, glutamine; Lac, lactate; His, histidine; Lys, lysine; Glu, glutamate; Glc, glucose; PC, phosphocholine; GPC, glycerolphosphocholine; MeOH, methanol; Cho, choline; sFA, saturated fatty acids; uFA, unsaturated fatty acids.

The S-plots corresponding to the above models are reported in Fig. S3, which shows the chemical shifts (i.e., the spectral position of each line in an NMR spectrum) of the metabolites that discriminate the classes. For further analysis, we selected signals that present the variable importance in the projection (VIP) > 1 and |*p*_corr_|≥ 0.6; they are indicated with black dots and identified in Fig. 3. Specifically, in the CN–AD model, AD patients show (Table 3) higher levels of glutamine (Gln) and lower concentrations of acetate (Ace), choline (Cho), isoleucine (Ile), leucine (Leu) and valine (Val), with respect to the CN group. The MCI-CN comparison found (Table 3) for MCI an increase of glucose (Glc), Gln, Ile, Leu, tyrosine (Tyr) and Val, and the reduction of Ace, lactate (Lac), glutamate (Glu), histidine (His) and lysine (Lys) (Fig. 3B, and Fig. S3B). In the SMD–AD model, an increased concentration of Ace, Cho, methanol (MeOH) and phosphocholine (Pc)/glycerolphosphocholine (Gpc) was found for SMD samples (Table 3), while unsaturated and saturated fatty acids (uFA and sFA, respectively) and Glc signals better characterized the AD class (Fig. 3C and Fig. S3C). Finally, the MCI–AD showed (Table 3) higher levels of alanine (Ala), Ile, Leu and Val for MCI, whereas the AD group exhibited an increased concentration of Glc, glyceryl lipids and Lac (Fig. 3D and Fig. S3D).

**Table 3:** Metabolite variation observed in the models established within the training set.

Model 1			Model 2		
**CN–AD**^**a**^			**CN–MCI**^**a**^		
**CN**	**Metabolite**	**AD**	**CN**	**Metabolite**	**MCI**
	**Gln**	**↑**		Glc, **Gln**, Ile, Leu, **Tyr**, Val	**↑**
**↑**	Ace, Cho, Ile, Leu, **Val**		**↑**	Ace, Lac, Glu, **His**, Lys	

Model 3			Model 4		
**SMD–AD**^**a**^			**MCI–AD**^**a**^		
**SMD**	**Metabolite**	**AD**	**MCI**	**Metabolite**	**AD**
	Ace, Cho, **MeOH**, Pc/Gpc	**↑**		**Glc**, Glyceryl lipids, Lac	↑
**↑**	**uFA**, sFA, Glc		**↑**	Ala, Ile, Leu, **Val**	

^a^Upward arrows indicate a higher concentration of the metabolites in the corresponding class with respect to the other one. Metabolites in bold present VIP > 1 and |p_corr_|≥ ± 0.6. Ace, acetate; Ala, alanine; Cho, choline; Glc, glucose; Gln, glutamine; Glu, glutamate; Gpc, glycerolphosphocholine; His, histidine; Ile, isoleucine; Lac, lactate; Leu, leucine; Lys, lysine; MeOH, methanol; Pc, phosphocholine; sFA, saturated fatty acids; Tyr, tyrosine; uFA, unsaturated fatty acids; Val, valine.

Differences in the concentration levels of the discriminant metabolites for the four models are reported in the S-line plots of Fig. S4A-D, which describe the loadings values as a function of the chemical shift. Since all buckets are normalized to the total spectrum area (see “Materials and methods”), the up and down peaks imply that concentration alteration does not depend upon dilutional effects.

### Validation of the models with an independent set

**Figure 4:**
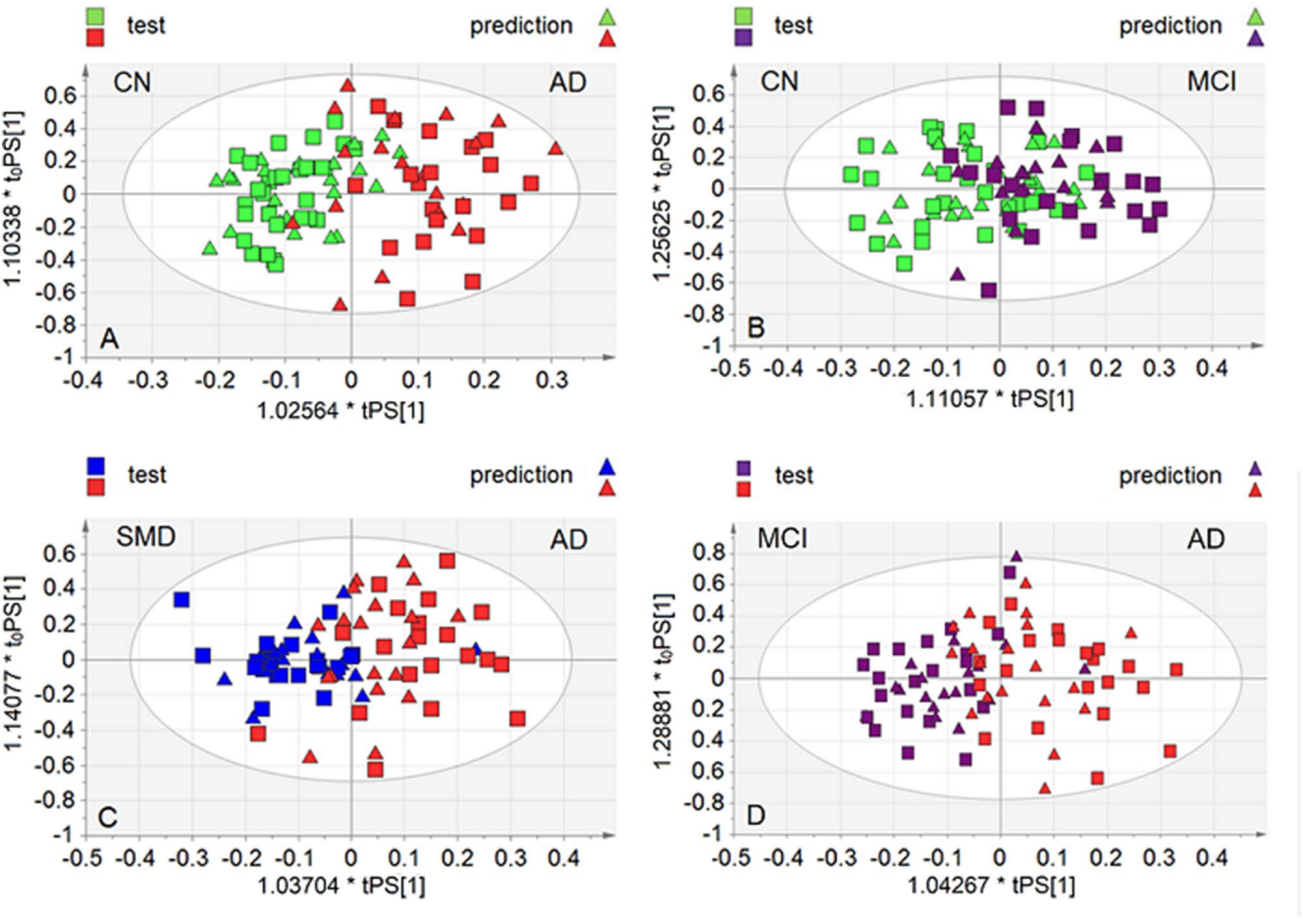
Predicted scores plot representing classification of the test set obtained with samples projection onto the OPLS-DA models assessed by the training set. Squares represent the training set samples (ts), while triangles refer to the validation set samples (ps, predicted set). (**A**), CN *vs.* AD; (**B**), CN *vs.* MCI; (**C**), MCI *vs.* AD; (**D**), SMD *vs.* AD.

The models’ performance was evaluated using a sample set not included in the model calculation. Specifically, we considered 81 new samples comprising 23 CN, 20 SMD, 19 MCI and 19 AD patients. They were projected onto the corresponding statistical model, and the results are displayed in Fig. 4. The OPLS-DA classifications of both training and predicted sets present a good class discrimination along the x-axis for CN–AD (Fig. 4A) and SMD-AD (Fig. 4C), while confirming the partial overlap for CN–MCI (Fig. 4B) and MCI–AD (Fig. 4D).

The performance of each model is shown in Table 4, together with the predictive parameters extracted from the corresponding confusion matrices. ROC curves were obtained taking into account the Y predicted response (Y_Pred_PS) generated by each OPLS regression. Y_Pred_PS is the Y value predicted by the model based upon the X block variables (resonance intensities at given ppm). The found AUC (area under the ROC curve) values were as follows: 0.99 for CN–AD, 0.88 for CN–MCI, 0.96 for SMD–AD, 0.97 for MCI-AD (Table 4), confirming that the test set well fits the built models. Furthermore, high sensitivity (true positive rate) values were observed for the four models (93, 88, 95 and 92%, respectively), while specificity (true negative rate) values reached 65% for CN–AD, 44% for CN–MCI, 45% for SMD–AD and 50% for MCI–AD. The lower values reflect the partial overlap observed in the corresponding models (Fig. 3), which most likely is an indication of the heterogeneous pathophysiology of the classes involved. Better values were obtained for precision (73, 61, 63 and 65%) and accuracy (79, 66, 70 and 72%); however, the presence of a between-class “uncertainty zone” requires further investigations on the transitional pathophysiology, to avoid false positive (FP) or false negative (FN) misclassification.

**Table 4:** Parameters summarizing the performance of each model on the test set.

Classification performance						
**OPLS-DA**	**ROC (AUC)**^***a***^	**Sensitivity (%)**^***b***^	**Specificity (%)**^***b***^	**Precision (%)**^***b***^	**Accuracy (%)**^***b***^	**CV-ANOVA p value**^***c***^
Model 1: CN–AD	0.99	93	65	73	79	0.003
Model 2: CN–MCI	0.88	88	44	61	66	0.01
Model 3: SMD–AD	0.96	95	45	63	70	0.001
Model 4: MCI–AD	0.97	92	50	65	72	0.008

^a^The area under the ROC curve (AUC) of the receiver operating characteristic (ROC) curve was calculated for each binary classifier in terms of the associated Y_Pred_PS values in predicting the class membership for samples of the test set. Y_Pred_PS is the Y value predicted by the model based upon the X block variables (resonance intensities at given ppm). An Y_Pred_PS value close to 1 would indicate that the subject is likely to belong to the class. An Y_Pred_PS value close to 0 would indicate that the subject is unlikely to belong to the class.

^b^Sensitivity, Specificity, Precision and Accuracy levels were obtained from the constructed Confusion Matrix combining the different fractions of true positive (TP), false positive (FP), true negative (TN) and false negative (FN) values for each classification.

^c^*p* value for the OPLS-DA reliability (*p* < 0.05).

### Identification of statistically significant biomarkers

To identify significant metabolites, ROC analysis and the corresponding AUC were calculated together with the Student’s *t* test. We selected metabolites showing both VIP > 1 and |*p*_*corr*_|> ± 0.6, namely, Gln, Glc, His, MeOH, Tyr, Val and uFA (Table S4). After, for each class comparison, we applied simple and multiple logistic regressions to investigate whether a single variable (*i.e.*, metabolite), or a combination of them, could improve classification of the different cognitive states.

**Figure 5:**
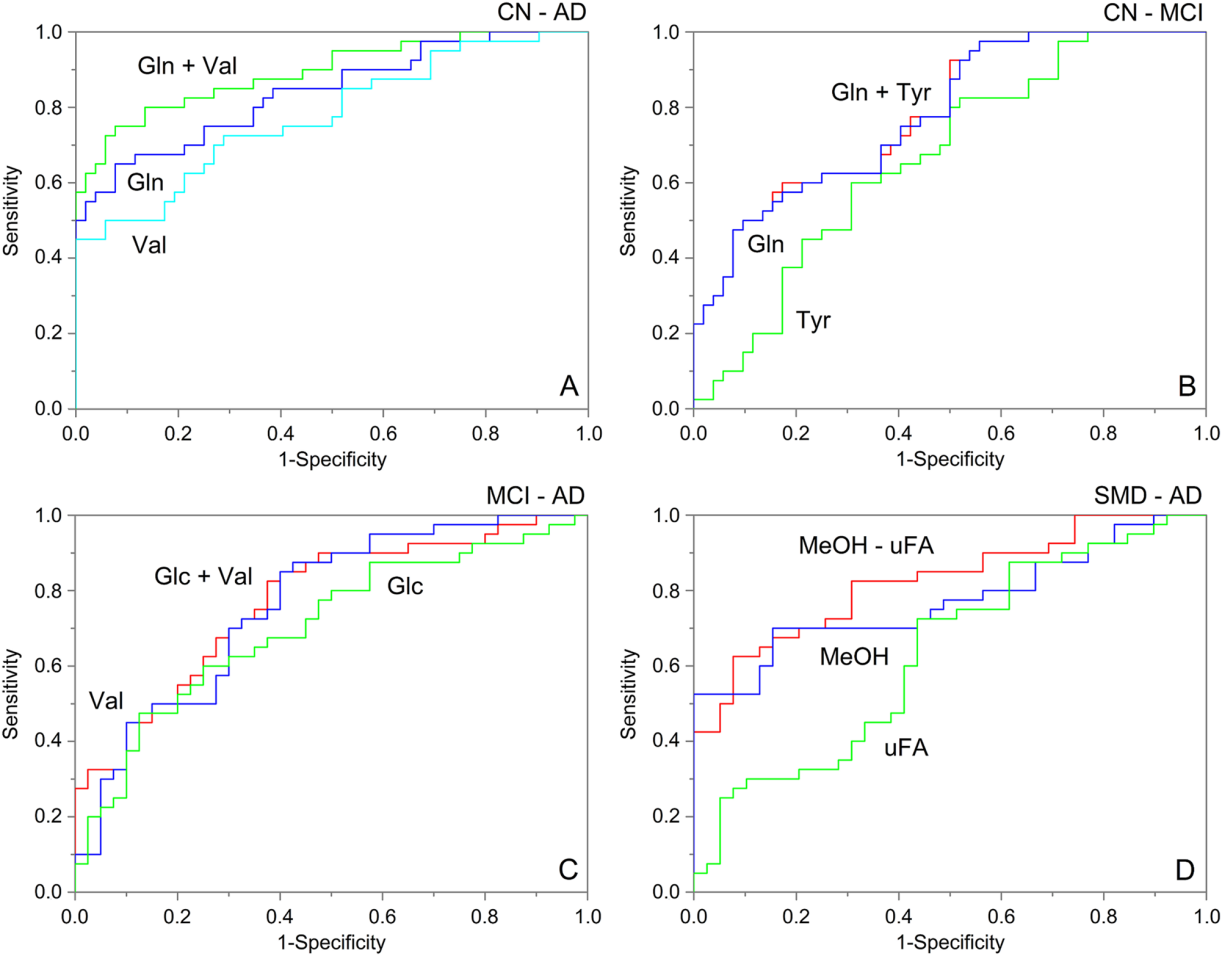
Simple and multiple logistic regressions of selected variables for each discriminating model: (**A**), CN *vs.* AD; (**B**), CN *vs.* MCI; (**C**), MCI *vs.* AD; (**D**), SMD *vs.* AD. Receiver operating characteristic (ROC) curve and the corresponding area under the ROC curve (AUC) of each classifier are reported together with the Student’s *t*-test applied on the normalized buckets concentration.

Specifically, for the CN–AD model (Fig. 5A) we selected Gln and Val. The corresponding ROC curves showed AUC values of 0.78 ± 0.05 for Val and 0.84 ± 0.04 for Gln, while for their combination it increases to 0.89 ± 0.03. The associated box-and-whisker plots (Fig. S5A) show that in AD Gln increases its concentration (left panel, *p* < 0.001), while Val decreases (right panel, *p* < 0.001). For the CN–MCI comparison (Fig. 5B), we considered Tyr, Gln and His. Their combination revealed no additional contribution with respect to each variable: Tyr + Gln resulted in the same AUC value as for the Gln variable alone (AUC = 0.78 ± 0.05), while Tyr alone showed a value of 0.66 ± 0.06. Similarly, AUC value for His + Gln equals the one related to the His alone (AUC = 0.78 ± 0.05; not shown). In this model, both Tyr (left panel) and Gln (right panel) present a higher concentration in MCI compared to CN (Fig. S5B, *p* < 0.05 and *p* < 0.001, respectively). No marked improvement was observed for the Glc–Val combination in the MCI and AD comparison (Fig. 5C), with AUC = 0.77 ± 0.05 for Glc + Val, whereas AUC = 0.76 ± 0.05 for Val and AUC = 0.71 ± 0.06 for Glc, separately. In AD, their concentration respectively increases (left panel) and decreases (right panel) with respect to MCI (Fig. S6A, *p* < 0.05 and *p* < 0.001, respectively). Finally, for the SMD–AD classification, we found a higher value AUC = 0.82 ± 0.05 for the combination of MeOH and uFA (Fig. 5D) with respect to separated variables (AUC = 0.77 ± 0.05 for uFA and AUC = 0.63 ± 0.06 for MeOH). In AD, their concentrations decreased (left panel) and increased (right panel) (Fig. 5B, *p* < 0.001 and *p* < 0.05, respectively).

Figure S7 shows the concentration levels and the ANOVA test significance for the selected metabolites in the four classes. In AD, the Glc concentration (panels A) is higher with respect to CN, SMD and MCI (*p* < 0.05), and can only differentiate AD from other classes, but no other discrimination can be obtained. MeOH and Val (panels D and F, respectively) present a lower concentration in AD. MeOH can discriminate AD from CN (*p* < 0.05) and SMD (*p* < 0.001), while Val separates AD from the other classes (*p* < 0.001). Tyr (panel E) is a useful marker to discriminate CN from MCI (*p* < 0.05), while Gln (panel B) shows a good discriminating power between CN and SMD (*p* < 0.05), and between SMD and AD (*p* < 0.05). Discrimination between CN and AD, and between MCI and AD, could also be obtained using Gln (*p* < 0.001). On the contrary, His presents a steady concentration decrease (panel C), with an efficient separation for CN and MCI (*p* < 0.05), as well as between CN and AD (*p* < 0.001), and between SMD and AD (*p* < 0.001).

Notably, the enrolled subjects presented statistically significant differences in age, BMI and education level both in the training (Table 1) and the test (Table 2) sets [differences in the Mini Mental State Examination (MMSE) are a direct indication of the AD evolution]. To account for their possible influence as covariates on measured metabolites and remove their potential effect on NMR variables, we performed ANCOVA for both sets. After means’ correction [*i.e.*, factoring out (excluding) the influence of such covariates], the *p*-value for the selected metabolites resulted < 0.05, with the difference between the metabolite levels in the (HS, SMD, MCI and AD) classes remaining statistically significant.

### Pathway topology analysis

**Figure 6:**
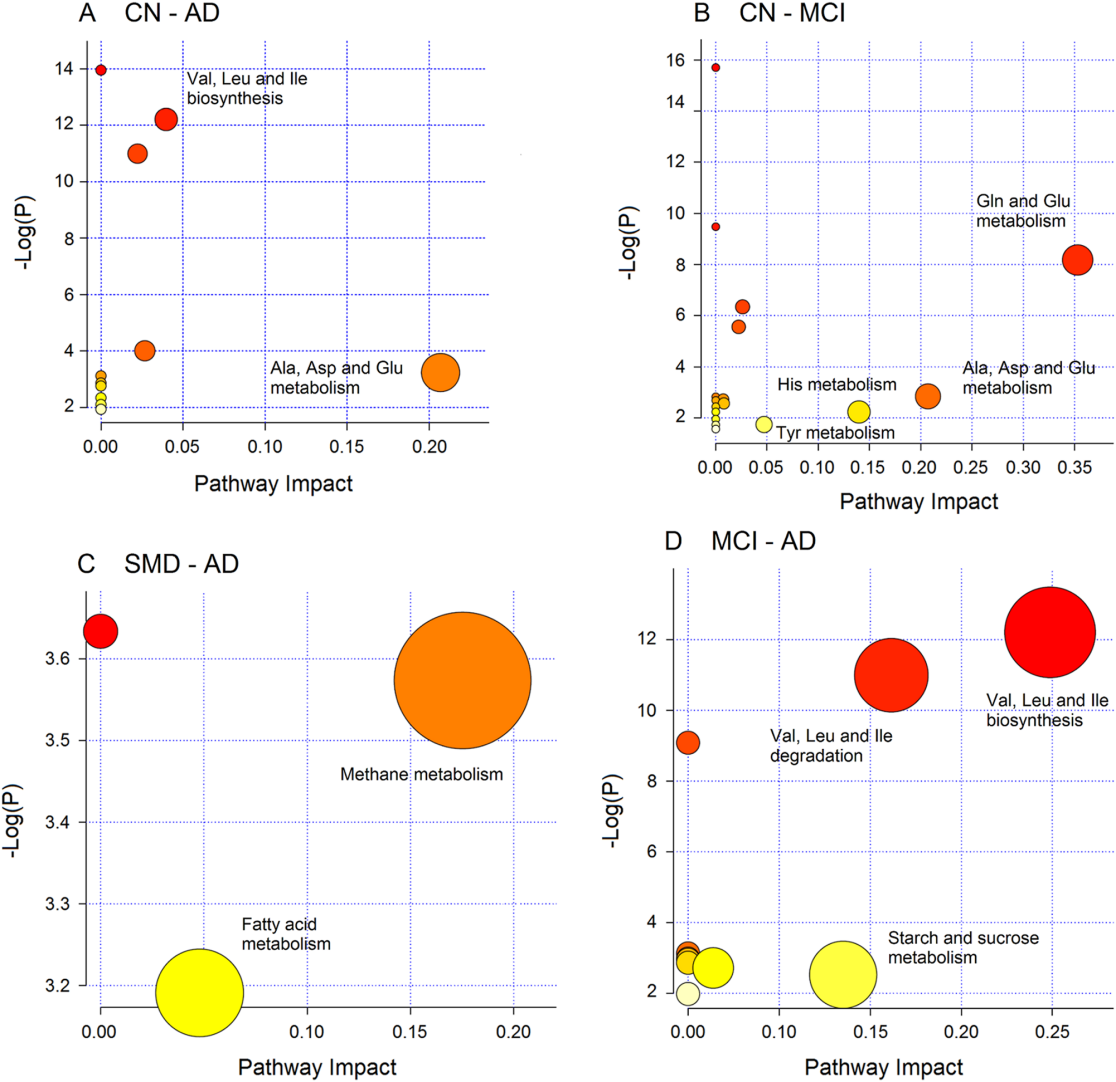
Pathway analysis overview showing the altered metabolic pathways associated with blood markers derived from discriminant class analysis: (**A**), CN *vs.* AD; (**B**), CN *vs.* MCI; (**C**), SMD *vs.* AD and (**D**), MCI *vs.* AD. For each class comparison, metabolites used presented both VIP ≥ 1 and |*p*_corr_|≥ 0.6. The most relevant networks are labeled considering the pathway impact and the *p* value.

The biological relevance of the data was evaluated by investigating the metabolic pathways that appear to be significantly dysregulated. In particular, we conducted pathway enrichment analysis, combined with pathway topological analysis, for each set of markers derived from the binary class discriminations. The found pathways are depicted in Fig. 6, which reports the impact of each pathway versus the *p* value. In the CN–AD comparison, among the 11 detected pathways, we inferred the Ala, Asp and Glu metabolism (*p* = 5.75 × 10^–4^; impact 0.23) as the most probable (Fig. 6A). For the CN and MCI comparison, amid the 17 identified pathways, we obtained Gln and Glu (*p* = 2.82 × 10^–4^; impact 0.36), Ala, Asp and Glu (*p* = 5.84 × 10^–3^; impact 0.22), and His (*p* = 1.05 × 10^–3^; impact 0.14) metabolisms (Fig. 6B). The SMD–AD model yielded 3 pathways, and the methane metabolism pathway (*p* = 2.81 × 10^–2^; impact 0.18) was the most probable (Fig. 6C). Finally, the MCI–AD comparison indicated, among the 10 detected pathways, Val, Leu and Ile metabolism (*p* = 5.00 × 10^–6^; impact 0.25), Val, Leu and Ile degradation (*p* = 5.00 × 10^–6^; impact 0.25), and starch and sucrose metabolisms (*p* = 5.00 × 10^–2^; impact 0.13) (Fig. 6D). Interestingly, the found pathways are all different for each comparison, implying that the physiopathology of SMD, MCI and AD is due to dysregulation of specific pathways that could be differently targeted and evaluated for diagnostic purposes.

By combining all markers found in the CN–AD continuum, we obtained 26 potential pathways, but Gln and Glu (*p* = 6.02 × 10^–11^; impact 0.29), Ala, Asp and Glu (*p* = 5.45 × 10^–9^; impact 0.21), His (*p* = 2.47 × 10^–5^; impact 0.14), and Tyr (*p* = 9.64 × 10^–3^; impact 0.05) metabolisms were the most relevant networks (Fig. S8), which correspond to those found for the CN–MCI model (Fig. 6B).

### Correlations

We found no correlation between metabolomic data and clinical analysis in any study group. In all groups, there was no correlation between found metabolites and comorbidity parameters, between metabolomic profiles and pharmacological therapies, and between metabolites and any of the anthropometric parameters of the experimental and validation sets (Tables 1 and 2).

## Discussion

We applied NMR-based metabolomics and PLS–DA statistical analysis to sera aiming at uncovering preclinical signatures of AD. We profiled 28 CN, 20 SMD, 21 MCI and 21 AD subjects as training set, and then validated the found models with a second independent cohort of 81 people, not included in the primary analysis. From NMR data, we obtained four statistically significant PLS–DA models, namely CN–AD (*p* = 0.004), SMD–AD (*p* = 0.002), CN–MCI (*p* = 0.039) and MCI–AD (*p* = 0.01). Clear class discrimination was observed for the CN–AD and SMD–AD models, while some overlap was present for CN–MCI and MCI–AD (Fig. 3B,D, respectively). The clinical spectrum of AD indicates that there is no definite cut-off point to discriminate intermediate grades (SMD and MCI), as well as normal aging and dementia. Furthermore, neuropathological studies have shown that patients with AD often have heterogeneous brain pathology and a considerable number of patients exhibits brain changes, indicating the interference of other concomitant neurodegenerative disorders^13^. Therefore, the intermediate diagnostic uncertainty in the models may reflect the physiological overlapping “gray-zone” and the pathological heterogeneity.

The blind test of an independent cohort confirmed the validity of the found models. CN–AD and SMD–AD showed an AUC of 0.99 and 0.96, respectively, while for the CN–MCI and MCI–AD models we calculated an AUC of 0.88 and 0.97, respectively. Sensitivity was high (from 88 to 95%) for the four models, while specificity reached 65% for CN–AD, 45% for SMD–AD, 44% for CN–MCI and 50% MCI–AD (Table 4). Interestingly, lower values of specificity were observed for CN–MCI (44%), SMD–AD (45%) and MCI–AD (50%) models, which all involve an intermediate class. Notably, for the CN-to-AD conversion, Casanova et al. reported a specificity of 65.7% for the sera of 93 patients of the Baltimore Longitudinal Study of Aging, and 36% for the sera of 100 patients from the Age, Gene/Environment Susceptibility-Reykjavik Study, while sensitivity was 51% and 47%, respectively^9^. Specificity values are in line with ours (Table 4), but our AUC and sensitivity values (99% and 93%) compared with 92% and 90% reported by Mapstone et al.^8^. The discrepancy could be related to methodological differences (they both used mass spectrometry) and the use of a cohort of different size, but the intrinsic heterogeneity of the pathology and the possible involvement of the gut-brain axis in neurological diseases in population from different countries should also be considered^14,15^. Interestingly, the data by Mapstone et al.^8^ and Casanova et al.^9^ referred to a panel of plasma phospholipids, while the parameters of our classification performance were based upon a panel of different metabolites (amino acids, glucose, fatty acids, etc.). This implies that, regardless of the panel used, discriminating metabolites are all affected by complementary “boundary conditions” that can influence the predictive parameters of the models.

With the term “uncertainty zone”, we identify a between-class overlap at molecular level, most likely due to concomitant factors^16^. First, intermediate and contiguous classes (for example, SMD and MCI) show overlapping pathophysiology with common molecular features, as subjects do not always progress to dementia, and some individuals revert to normal cognition or remains stable. Second, mixed pathologies are frequently observed in AD, therefore showing commonalities with molecular aspects of other forms of dementia. Third, the correct threshold values for metabolites specific for each class are unknown^16^. Consequently, the biomarker-based diagnosis currently lacks specificity, and the derived phenotypes and endotypes have not prognostic value (*i.e.*, likelihood of progression from SMD to MCI, and to AD)^16^.

The CN–MCI and MCI–AD models present a specificity that compares with the reversion rate of MCI (towards CN, ranging from 30 to 50%), and the MCI progression rate (towards AD, comprised between 4 and 40%), with two-to-five year follow-up^17^. In addition, magnetic resonance imaging evaluation of the structural brain changes confirms that 43% of the stable MCI subjects has an AD-like pattern of atrophy, while 57% of the stable MCI subject had a CN-like pattern^18^.

Since SMD may evolve into MCI in a 13-month intervalwith a percentage between 5.6 and 18.9%^19^, and *ca.* 62% of subjects with mental decline do not disclose SMD^20^, the reduced sensitivity of the SMD–AD model appears to be justified^21^. This suggests that, with respect to molecular classification, the clinical classes could be unstable presenting different evolution further depending on the heterogeneous nature of SMD/MCI^22^. As an indirect confirmation, our SMD–MCI model was statistically non-significant (*p* = 0.68).

Accordingly, model parameters involving MCI and SMD probably reflect the heterogeneity of the states, as suggested by the values of precision (repeatability) and accuracy (trueness) (Table 4), which mirror how well the method performs. It is worth mentioning that—different forms of neurodegeneration, although being characterized by dissimilar manifestations, present similar pathophysiological and clinical processes^23^. The above data strongly suggest that the “uncertainty zone” originating from overlapping pathophysiological features in the AD process generates a partial similarity at molecular level. From the discriminating metabolites, we extracted the altered pathways that are statistically relevant in the progress of AD. Interestingly, they show that although the pathway Val, Leu and Ile biosynthesis is altered in CN–AD and MCI–AD models, and Ala, Asp and Glu metabolism is affected in CN–AD and MCI–AD comparisons, they present different impact percentage (*x* axis) and *p* parameters (*y* axis) in the different models (Fig. 6). This would suggest that the altered pathways derived even in the presence of overlapping pathophysiological features may become a more sensitive target in the CN-to-AD evolution, and be used to define specific molecular characteristics that could reduce the uncertainty in the classification models^16^.

### CN–AD

For CN–AD, serum metabolite profiling revealed significant perturbations in amino acid metabolism pathways involved in neurotransmission, namely Ala, Asp and Glu metabolism, and Val, Leu and Ile biosynthesis (Fig. 6A). The AD patients, compared to CN, showed higher levels of Gln and lower concentrations of Ace, choline, Ile, Leu and Val (Fig. 3A and Fig. S3A).

Bloodstrem Gln is basically produced by muscles, but this can be safely excluded as the enrolled AD patients were 79.1 ± 6.4 years of age in the training set (Table 1), and 77.5 ± 7.4 in the test set (Table 2). Since 90% of the Gln pool is generated by the neurotransmitter Glu taken up into astrocytes^24^, the Gln excess may be due to imbalance in the Glu–Gln conversion therefore reducing the availability of neurotransmitter Glu^25,26^. Considering the CSF leakage into the blood, also favored by the blood–brain barrier damage in AD^4–6^, we postulate that the observed Gln increase for AD with respect to CN may suggest an increase of Gln in the brain. In AD, inflammation induces oxidative stress, and a systemic inflammatory response can amplify the Gln need of the immune cells, generating a Gln flow from the brain^27^.

Ace, mainly produced by intestinal flora, can easily enter the brain from plasma. From the brain, Ace can revert back to plasma or go into astrocytes^28^, undergoes conversion to acetyl CoA and enters the TCA cycle, while a little quantity is involved into lipid pathways. Finally, it is integrated into amino acids, primarily Glu and Gln. As such, Ace reduction in AD patients with respect to CN subjects could be part of the molecular mechanisms responsible for the Gln increase, as they both act in astrocytes, where Gln is produced. Ace, one of the short-chain fatty acids (SCFAs), regulates various leukocytic acticities, and seems to influence leukocyte mobility to the inflammation foci^29^. Furthermore, according to the mevalonic acid pathway, Ace units produce cholesterol that that can activate the NLRP3 inflammasome, which significantly affects the AD pathogenic process. In fact, in an animal model, its inhibition efficaciously avoided spatial memory loss and reduced Aβ precipitation^30^.

Cho is a precursor of the neurotransmitter acetylcholine (of which Ace is a precursor), Pc (a cell membrane constituent), and glycerophosphocholine (a cerebral osmolyte). Cho changes are generally associated with cholinergic deficiency in human brain of MCI and AD^31^, as observed in both necropsy brain samples and CSF, and are thought to be responsible for the decline in learning and memory abilities^32^. The reduction of Ace could also bring about a decrease of acetylcholine so as to compromise the neurotransmitter activity.

Impairment in the Val, Leu and Ile (branched-chain amino acids, BCAAs) biosynthetic pathway could reduce their concentrations in AD. They are sources of energy and provide nitrogen for neurotransmitter synthesis. BCAAs are metabolized via transamination by the branched-chain aminotransferases, which are overexpressed in the brain of AD patients. Their overexpression leads to alterations in BCAA metabolism, which affect neurotransmitter synthesis and may contribute to the pathogenic mechanisms observed in AD^33^. Furthermore, Val, Leu and Ile are also linked to behavioral changes, and can induce mental retardation and neurological degeneration^34^. The serum level of Val has been reported to be reduced in AD and Huntington’s disease patients^35^.

Leu is a key regulator of the mechanistic target of rapamycin complex 1 (mTOR), which regulates the aging rate across invertebrates and mammals, and yields significant neuroprotective effects, improving cerebrovascular and cognitive function in mouse models of AD^36^. It was suggested that mTOR activation is significantly involved in blood–brain barrier (BBB) misfunctioning in AD, and that rapamycin and/or their analogs could be utilized to restore BBB integrity^36^. As such, the Leu reduction observed in AD, with respect to CN, could participate in the impairment of the mTOR altering the BBB and the molecular functioning of the cerebrovascular system^36^.

Alteration of Leu concentration also affects the Gln–Glu cycle via the so-called Leu–Glu cycle^37^. In the astrocyte compartment, through transamination, Leu donates its amino group to α-ketoglutarate to give rise to Glu and start the cycle, and its reduction may impair the Gln–Glu cycle in AD.

### CN–MCI

The main altered pathways in CN–MCI model were Gln and Glu metabolism, Ala, Asp and Glu metabolism, His metabolism, and Tyr metabolism (Fig. 6B). In MCI we found the predominance of Glc, Gln, Val, Ile, Leu and Tyr, and the reduction of Ace, Lac, Glu, His and Lys (Fig. 3B and Fig. S3B).

The human brain is almost completely dependent on Glc (and pyruvate) metabolism to meet energy demand. The increased concentration of Glc detected in MCI patients suggests an altered Glc metabolism with respect to CN^38^, as energy deficiency in the neuronal production due to modified rate of carbohydrate catabolism has been frequently reported for AD^39^. During the AD progression, accumulated β-amyloids inhibit Glu uptake through diminished Glc intake, altering Glu–Gln metabolism as shown by an increase in the concentration of Gln^40^, as here observed for MCI. The imbalance in the Glu–Gln conversion may again justify the increase/decrease of Gln/Glu we observed, and the reduced availability of neurotransmitter Glu, may cause memory loss in MCI subjects.

As for the CN–AD model, accumulation of Val, Leu and Ile in MCI could be an indication of deficiency in the BCAAs degradation system, with the implications above described. Furthermore, the increase of Leu in MCI could be an attempt to activate the mTOR system to stimulate neuroprotective effects^36^, and/or restore the Glu concentration^37^ in MCI.

His is essential for the brain synthesis of histamine. Increased brain His and histamine, especially in the hypothalmus, have been shown to occur in AD patients^7^. His, via its imidazole ring, is an antioxidant compound, and its concentration reduction in MCI compared with CN could be related to the oxidative stress associated with neurodegeneration. In fact, a decrease in imidazole containing amino acids has been observed in plasma, urine and CSF of AD patients^41^.

Tyr derives from Phe, and can generate dopamine, epinephrine, noradrenaline, and norepinephrine, all involved in neurotransmission. Its brain concentration can be increased by oral consumption, but peripheral Tyr absorption and metabolism may change with aging^42^. In humans, Tyr administration potentiated central catecholamine synthesis, which is also important for working memory^43^. Disturbance of Tyr pathway has recently been reported for plasma samples of AD patients in a comparison with CN subjects^44^.

Lys is involved in the l-carnitine synthesis, which transports fatty acids to mitochondria to produce energy. In fact, carnitine concentration was reduced in the CSF of MCI and AD with respect to control subjects^45^. Finally, it has been shown that malfunctioning of mitochondria and deficiency of the respiratory complexes are involved in neurodegeneration^46^.

Lac is crucial for energy metabolism and formation of memory^47^, and is a marker of acute inflammation^48^. Lac concentration was reduced in Aβ_25–25_-treated rat model of AD, while MCT2 (one of the monocarboxylate transporters that promote Lac transport into neurons) was reduced in the AD brain. This suggests diminished supply of energy substrate and reduced neuronal Lac uptake, and points to reduced metabolism in the AD brain^49^. Altered level of Lac has been reported for CSF samples from AD patients, which is paralleled by higher pyruvate levels^50^.

### SMD–AD

In the SMD–AD comparison, the main altered pathways are methane metabolism and fatty acid metabolism (Fig. 6C). In AD, we found increased uFA and sFA and Glc, and a decreased Ace, Cho, MeOH and Pc/Gpc moieties (Fig. 3C and Fig. S3C).

The dysregulation of methane metabolism and the reduced concentration of MeOH in AD may indicate an involvement of the human microbiota in the SMD-to-AD evolution. In fact, *Methanosphaera stadtmanae*, a methanogenic species isolated from the human colon, is implicated in the reduction of MeOH to methane with H_2_, and the synthesis of ATP^51^. In a rat model, in which focal ischemia and reperfusion were used to induce brain oxidative stress, H_2_ suppressed brain injury and its progressive damage because of its potent antioxidative, antiapoptotic and anti-inflammatory activities^52^. Interestingly, methanogens increase progressively with age in the human intestinal tract, reaching their highest concentrations in the elderly^53^. Therefore, it is tempting to speculate that dysregulation of the methanogenic metabolism could modifies H_2_ availability, affecting its neutralizing action against brain reactive oxygen species.

Furthermore, the methane metabolism, via methane, MeOH and formaldehyde, generates formate and then serine, which is important in neurological and psychiatric disorders^54^. It is an essential precursor of sphingolipids, which are also involved in lipid metabolism. In particular, the level of triglycerides has been associated with the serine metabolism^55^, which could establish a link between methane metabolism and lipid involvement in AD (vide infra).

Interestingly, modulation of gut microbiota by administrating nonabsorbable antibiotic improved the performance on a cognitive task in patients affected by hepatic encephalopathy and MCI^56^. Moreover, variations in gut microbiota was found in Parkinson’s disease (PD) patients, who showed a 78%-reduction of *Prevotellaceae* in the feces of PD patients with respect to controls^57^.

The brain metabolism of FA is substantially altered in patients with different AD grading, and FA have been implicated in AD progression^58^. Additionally, high levels of free FA favor amyloid deposition and tau hyperphosphorylation, which are involved in the AD pathogenesis^59^. Dysregulation of sFA metabolism is present in MCI plasma and in AD CSF, with FA omega oxidation observed in the plasma of AD patients^7^. The Pc decrease we observed in SMD, and the alterations in sFA and uFA, indicate a possible membrane destabilization related to imbalance in the levels of sFA/uFA, which are part of the phospholipids’ structure^60^.

Cho variations are generally linked to cholinergic deficiency in MCI and AD human brain^31^. Decreased Cho moiety (including Gpc and PE), together with the reduction of Ace, the precursor of acetylcholine, could compromise the neurotransmitter cycling in AD with respect to SMD. In addition, the glyceryl lipids diacylglycerols (see below) are produced from phosphatidylcholines in the synthesis of sphingomyelins via the transfer of the Pc headgroup to a ceramide^61^, which could affect the Pc level and be involved in the production of ceramide for the NLRP3 activation^30^.

### MCI–AD

In the MCI–AD comparison, the main pathways involved were Val, Leu and Ile biosynthesis, Ala, Leu and Ile degradation, and starch and sucrose metabolism (Fig. 6D). Furthermore, in AD an increase of Glc, Lac and glyceryl lipids, and lower levels of Ala, Ile, Leu and Val were detected (Fig. 3D and Fig. S3D).

Ala is a downstream product of taurine, which is found at high concentrations in the mammalian brain with neuroprotective and neurotrophic roles. Taurine can inhibit β-amyloid neurotoxicity^62^, and it is reduced in AD sera^63^. Therefore, lower Ala in AD would imply impairment in the taurine activity with respect to MCI. In the Ala, Asp and Glu pathway, Ala is downstream of the neurotransmitter *N*-acetylaspartate, whose concentrations in cortical gray matter decrease with age^64^.

Glyceryl lipids are the esterification of fatty acids to the hydroxyl groups of glycerol. It has been reported that plasma^65^ and the frontal cortex^66^ of AD patients show higher levels of diacylglycerols (DAGs). DAGs are critical components of membranes, are involved in lipid metabolism and are essential for lipid-based signaling^61^. In particular, cell membrane breakdown is involved in acute and chronic neurodegeneration because it alters permeability, fluidity and ionic homeostasis^67^. Finally, high concentration of lipids is one of the key vascular factors for AD pathogenesis^68^. Therefore, it appears that increased levels of glyceryl lipids DAGs characterize the AD evolution (we found glyceryl lipids increased in AD with respect to MCI), reaching high discriminating levels in AD.

Starch and sucrose metabolism belongs to the carbohydrate metabolism, which allows the cells to access energy. A dysregulation of such metabolism was found comparing genes differently expressed in AD with the control expression in the human temporal cortex^69^. The increased concentration of Glc in AD profiles compared to MCI could be linked to this. The AD incidence is augmented in subjects with diabetes^70^, and in those with higher Glc levels^71^. The glycation reaction between Glc and glycolysis metabolites with cellular components (DNA, lipids and proteins) produces advanced glycation endproducts (AGEs), which are reported in the early AD stages, and may take part in the development of neurofibrillary tangles and senile plaques^72^. Furthermore, in AD brain, the macrophage migration inhibitory factor (MIF) undergoes glycation and oxidation, which prevent the MIF stimulation of glial cells^73^. Since it also act as immunomodulator and insulin regulator, it is hypothesized that modified MIF relates hyperglycemia, oxidative stress and altered AD innate immune system^73^.

In parallel, defects in brain Glc homeostasis are reported to be inherent in AD pathological mechanisms, and may start many years prior to the clinical manifestations’ onset. Furthermore, in AD, brain areas exposed to senile plaques and neurofibrillary tangles present considerably increased levels of Glc in tissues, which are linked to greater severity of both plaque deposition and tangles pathology^38^. More importantly, higher levels (i.e., last measured fasting plasma Glc concentration) as well as greater increases over time in plasma fasting Glc are associated with higher brain tissue Glc concentrations, confirming the exchange of metabolites between blood and brain^38^.

The number of subjects involved in the study could be a possible shortcoming. However, we considered all the four stages of the AD continuum and enrolled a blind control set of patients including again the four classes. We obtained robust group discrimination and a diagnostic performance in line with previous studies^8,9^, ruling out overfitting of the models that could affect the results. An overfitting model usually yields a poor performance as it amplifies minor data fluctuations.

The objective of this study was to uncover AD prodromal signs in blood, a minimally invasive biomatrix, since a diseased central nervous system can drive a peripherally detectable biosignature^74,75^. The finding that in AD patients plasma and CSF present overlapping perturbed metabolic pathways^7^, strengthens the relationship between peripheral blood and neurodegenerative brain changes. Genetically engineered mouse models, although helpful for understanding AD pathogenesis and therapeutics, might present metabolic differences that could not include all the human AD features. In fact, it has been reported that the metabolic profile of mice and humans’ brains diverges consistently with their genetic/evolutionary lineage^76^. Therefore, it is important to investigate also human biomatrices to gain a clinically useful description of metabolite alterations induced by AD.

One problem that should be addressed relates to the reported variable and often contrasting results observed with the proposed panels of biomarkers. This is certainly linked to methodological aspects (the different technical methods used, the number of enrolled subjects, samples’ conservation, etc.). However, evidence is accumulating that AD is a heterogeneous and multifactorial disorder, resulting in different phenotypes/endotypes^77^, each characterized by different pathological and molecular mechanisms, prognosis, and therapy response. As such, the “uncertainty zone” observed in our models could be a manifestation of the AD multifactoriality, which should be addressed when analyzing blood biomarkers. For example, the six-metabolite panel we validated from peripheral blood was able to classify SMD, MCI and AD with sensitivity values ranging from 88 to 95% and ROC values from 0.88 to 0.99. Nonetheless, the values of specificity (44 to 65%), accuracy (66 to 72%) and precision (61 to 73%) attributable to the heterogeneity of SMD and MCI in the progression towards AD may limit the applicability of blodd-based biomarkers.

From the discriminating metabolites, we extracted the altered pathways that are statistically relevant in the progress of AD. Interestingly, they show that although the pathway Val, Leu and Ile biosynthesis is altered in CN–AD and MCI–AD models, and Ala, Asp and Glu metabolism is dysregulated in CN–AD and MCI–AD, they present different impact percentage (*x* axis) and *p* parameters (*y* axis) in the different models (Fig. 6). This would suggest that the altered pathways derived even in the presence of overlapping pathophysiological features is a more sensitive target in the CN-to-AD evolution, and could be used to define specific molecular characteristics that could reduce the uncertainty in the classification models^16^. Interestingly, from all metabolites found in the CN-to-AD continuum, we found that the most relevant networks (Fig. S8), correspond to those found for the CN–MCI model (Fig. 6B). This suggests that MCI represents the most relevant metabolic breakdown, pointing to those pathways as the primary targets for prodromal and preclinical investigations.

It is concluded that blood-based biomarkers require a careful evaluation for the assessment of early neurodegeneration because of the molecular overlap. A step forward would be to define the molecular threshold of biomarkers that characterize the metabolic phenotype/endotype of each class. However, the pathological heterogeneity suggested by clinical data from which our results seem to derive, rule out a “linear” molecular evolution of the pathology, pointing to the presence of overlapping “gray-zones” due to the reciprocal interference of the intermediate stages.

## Materials and methods

### Subject population and sample collection

Two hundred fifty participants were consecutively recruited from the Centre for Research and Training in Medicine for Aging (CeRMA), University of Molise (Italy). Of them, 50 subjects were excluded, and 29 sera gave NMR spectra unsuitable for analysis, amounting to a total of 171 subjects considered in the study. Patients with Alzheimer’s clinical syndrome were diagnosed according to National Institute on Aging/Alzheimer’s Association (NIA–AA) criteria^78^. MCI subjects showed both subjective and objective memory impairment, SMD participants presented only memory complaints with a normal score on the memory tests, and CN showed neither subjective nor objective memory impairment (see Supplementary data for details). Depression at screening was assessed with the Geriatric Depression Scale (GDS)^79^, and participants with a GDS score of 6 or more were considered depressed and excluded from the study. The patients on treatment with cerebro-active drugs underwent a washout period of at least 14 days before assessment. Subjects were sampled including risk factors for AD, as well as different types of medication used to treat comorbidities in AD (see Supplementary Information for details).

After evaluation of the inclusion/exclusion criteria and acquisition of NMR spectra of serum samples (see Supplementary Information), we excluded 79 partecipant (49 CN, 13 SMD, 9 MCI and 8 AD) samples. Therefore, a total of 171 subjects were considered in the study. Subjects were randomly allocated in two groups: the first (the training group, Table 1) comprised 90 patients and was used to generate the statistical models based on NMR data; the second (the test group, Table 2), not considered for the primary analysis, included 81 patients, and was used as a control set to verify blindly the models’ reliability. The first 90 subjects included: 28 CN (15 F, 13 M), 20 SMD (13 F, 7 M), 21 MCI (13 F, 8 M), and 21 AD (14 F, 7 M). The same four classes were present in the test set, which included 23 CN (10 F, 13 M), 20 SMD (13 F, 7 M), 19 MCI (13 F, 6 M), and 19 AD (13 F, 6 M) (Fig. 1). The patient distribution was unknown to the NMR and statistical analysis people.

The study was approved by the Regional Health Autority of University of Molise. Written informed consent was obtained from subjects or caregivers, who were completely informed about the procedures. The ethical principles of the Declaration of Helsinki, and the national and international guidelines for human research were followed.

Venous blood was collected with standard clinical procedures between 7:30 and 8:00 am after an overnight fasting of *ca.* 12 h. For collection we used vacutainer serum tubes (Becton & Dickinson, Milan, Italy); samples were coagulated at room temperature for 10 min, and then a 10-min centrifugation at 3,000 g was applied. Supernatants, frozen in liquid nitrogen, were packed with dry ice, and sent by courier to the NMR laboratory. Upon arrival, they were stored at − 80 °C until measurements.

### Sample preparation

Stored sera were rapidly defrosted and 330 µl of every serum were diluted with 300 µl of saline solution 0.9% sodium chloride in water (pH 7.4), and 70 µl of D_2_O, and were transferred into 5-mm NMR tubes for acquisition.

### NMR spectra acquisition

Two^1^H-NMR spectra were acquired for each serum sample on a Bruker Avance III–600 MHz spectrometer (BrukerBioSpin GmbH, Rheinstetten, Germany), equipped with a TCI CryoProbe fitted with a gradient along the Z–axis, at a temperature of 27 °C: (1) a standard one-dimensional (1D) proton spectrum; and (2) a T_2_-edited 1D spectrum where signals from proteins and others macromolecules were attenuated with use of short spin–spin relaxation times employing the Carr–Purcell–Meiboom–Gill (CPMG) pulse sequence with water presaturation^80^, and using a fixed inter echo delay to eliminate diffusion and J-modulation effects. These spectra were analyzed with multivariate analysis. Moreover, two-dimensional (2D) clean total-correlation spectroscopy (TOCSY) and heteronuclear single quantum coherence (HSQC) experiments were acquired to identify metabolite signals. Spectral signals were referenced to the lactate doublet assumed to resonate at δ = 1.33 ppm for^1^H, and δ = 20.76 ppm for^13^C, as added reference compounds can bind nonspecifically to serum albumin and others proteins thus affecting reference chemical shift and peak resolution (see Supplemental Information for details).

### Statistical analysis

#### Power analysis

To determine sample size, we resorted to a strategy we reported previously^81^. Since for projection methods like OPLS analysis no standard methods exist, the power of the analysis, our study was considered as a pilot study for which no a priori power analysis was possible. Our results were used to backward calculate the power of our analysis since biomarkers and their variations that could discriminate classes were unknown before analysis. The parameters 1-α and 1-β were varied from 95 to 99.9%, and from 80 to 99.9%, respectively, and the percentages of accuracy obtained in the validation tests (vide infra) were used for Unexposed and Exposed subjects. For 1 − α = 95% and 1 − β = 80%, we obtained 18 ± 2 CN, 16 ± 3 SMD, 15 ± 2 MCI and 17 ± 2 AD; while for 1 − α = 99.9% and 1 − β = 99.9%, we obtained 19 ± 3 CN, 18 ± 2 SMD, and 17 ± 3 for MCI and AD. The number of the subjects used in this study for each group is in agreement with (or is larger than) those obtained with backward analysis, although typical values are 1 − α = 95% and 1 − β = 80%, and a value of 99.9% for both is an extreme setting.

#### Spectral and multivariate analysis

Spectral and multivariate analysis was carried out as reported^81^. Using AMIX 3.6 software package (BrukerBioSpin GmbH, Rheinstetten, Germany), we performed an automatic data reduction to integrated regions (“buckets”) of 0.04 ppm each. The procedure was applied to the spectral region between 0.04 and 9.40 ppm of all spectra. The region containing the residual irradiated water resonance (4.72–5.10 ppm) was not considered, and normalization to the total spectrum area was achieved for the integrated section. To discriminate according to their NMR profiles, we applied multivariate statistical analysis using projection methods. We next imported the obtained data format (X matrix) into SIMCA-P + 14 package (Umetrics, Umeå, Sweden), and carried out PCA and OPLS-DA^82^. We initially analyzed the first batch of 90 samples and used it to create the training set that was employed to predict the second batch of 81 samples used as blind test set. As data pre-treatment for PCA, we applied mean-centering since all spectra did not show large differences. Then, for OPLS-DA, Pareto scaling turned out to be suitable to better appreciate clusters and the spectral variables that influenced class distribution. PCA was conducted to decrease data dimensionality and to uncover potential clusters of the CN, SMD, MCI and AD classes. After assessment of class homogeneity for every class, we applied supervised OPLS-DA, where a matrix Y comprising dummy variables was used. To build predictive models, we performed supervised regressions on two groups at a time. Visualization was achieved through scores plots, which also highlighted putative markers useful for classification. OPLS-DA models were validated by an internal iterative cross-validation with 7 rounds permutation test response (800 repeats), and CV-ANOVA (ANOVA testing of Cross-Validated predictive residuals) The models built with the training set were to classify the test set patients. Selected isolated signals with |*p*_corr_|≥ 0.6, VIP > 1 (Variable Importance in the Projection) were then considered for Student’s *t*-test and ANOVA test with Bonferroni correction, after ANCOVA (Analysis of Covariance) data adjustment for the statistically significant covariates (namely, age, BMI and education level) in both training and test sets (Tables 1 and 2), elaborated with the OriginPro 9.1 software package (OriginLab Corporation, Northampton, USA) and R software [R core team (https://www.r-project.org/).

#### ROC curve analysis

ROC curves were constructed reporting sensitivity (*y*-axis) *vs.* 1-specificity (*x*-axis), and area under the ROC curve (AUC) was calculated for each OPLS-DA model directly on Y_pred_PS values in SIMCA-P + 14. Next, ROC curves on selected signals were obtained importing normalized buckets values in the OriginPro package. ROC curves accounting for the combination of multiple metabolite responses were obtained with multilinear logistic regression (MLR) included in the Origin software. Values of AUC > 0.97, 0.93–0.96, 0.75–0.92 and 0.6–0.74 are interpreted as “excellent,” “very good,” “good” and “reasonable,” respectively. An AUC of 0.5 indicates a test with no discriminatory power.

#### Pathway analysis

Relevant metabolites highlighted from the statistical models were addressed to pathway analysis in order to identify the most significant metabolic networks involved in the conditions under study. Metabolite identifiers (KEGG code) together with their corresponding normalized bucket intensities were used for pathway enrichment analysis and pathway topological analysis, employing the MetaboAnalyst platform (www.metaboanalyst.ca). We selected the *Homo sapiens* library, and Global Test and Relative Betweenness Centrality were chosen for pathway enrichment analysis and pathway topological analysis, respectively.

## Supplementary information

Supplementary Information
