# Supplementary material for: Blood biomarkers indicate that the preclinical stages of Alzheimer's disease present overlapping molecular features
Source: Sci Rep. 2020 Sep 24;10:15612. doi: 10.1038/s41598-020-71832-y (PMC7515866; doi:10.1038/s41598-020-71832-y)
Supplement: Supplementary file 1 — Supplementary Information [file 41598_2020_71832_MOESM1_ESM.pdf]

Supplementary Material

Blood biomarkers indicate that the preclinical stages of Alzheimer's disease present overlapping molecular features

Alfonso Di Costanzo[1], Debora Paris[2], Dominique Melck[2], Antonella Angiolillo[1], Gaetano Corso[3], Mauro Maniscalco[4], Andrea Motta[2]

[1]Centre for Research and Training in Medicine for Aging, Department of Medicine and Health Sciences "Vincenzo Tiberio", University of Molise, I-86100 Campobasso, Italy.

[2]Institute of Biomolecular Chemistry, National Research Council, 80078 Pozzuoli (Naples), Italy.

[3]Department of Clinical and Experimental Medicine, University of Foggia, 71122 Foggia, Italy.

[4]Pulmonary Rehabilitation Unit, ICS Maugeri SpA SB, Institute of Telese Terme, 82037 Telese Terme (Benevento), Italy.

A. Di Costanzo and D. Paris contributed equally to this paper.

Correspondence to Andrea Motta: andrea.motta@icb.cnr.it and/or Debora Paris: debora.paris@icb.cnr.it

Online supplementary material

The Methods section details subject classification and NMR data acquisition. Fig. S1 shows the PCA scores plots of all training and validation samples used in the study, aiming at detecting possible outliers. Fig. S2 depicts representative 1D NMR spectra CN, SMD, MCI, and AD subjects. Fig. S3 reports the S-plots (with the chemical shifts of the metabolites that discriminate the classes) corresponding to the statistically relevant models. Fig. S4 depicts the S-line plots between 8.7 and 0.5 ppm corresponding to the Fig S3A-D models. Fig. S5 shows the box-and-whisker plots of the concentration levels of the discriminating metabolites for the CN-AD and the CN-MCI models. Fig. S6 represents the box-and-whisker plots of the concentration levels of the discriminating metabolites for the MCI-AD and the SMD-AD models. Fig. S7 displays the box-and-whisker plots of the concentration levels and the ANOVA test significance for selected metabolites in the CN, SMD, MCI and AD classes. Fig. S8 depicts the pathway analysis overview of the altered metabolic pathways associated with all blood markers derived from discriminant class analysis involving CN, SMD, MCI and AD. Table S1 describes the parameters of the PCA model for each sample class of the training set. Table S2 reports the 1H-NMR assignments of the metabolites identified in the studied human samples. Table S3 lists the diagnostics parameters of the statistical significant OPLS-DA models in the training set. Table S4 defines the variables (metabolites) selected from each OPLS-DA statistical model for single and multiple ROC curve analysis.

Methods

Subjects

We consecutively recruited 250 study partecipants from the Centre for Research and Training in Medicine for Aging (CeRMA), University of Molise (Italy). The Mini Nutritional Assessment (MNA)[1] showed that all participants were well nourished (MNA score > 23.5), with the exception of 11 AD patients who were at risk of malnutrition (MNA score between 19 and 23), but not malnourished. Patients with Alzheimer's clinical syndrome were diagnosed according to National Institute on Aging / Alzheimer's Association (NIA-AA) criteria[2] and fulfilled the criteria for "probable AD with documented decline" category. They presented Mini Mental State Examination (MMSE) score < 24[3] and Clinical Dementia Rating (CDR) score > 0.5[4]. Subjects with amnestic MCI met the NIA-AA diagnostic criteria for MCI due to AD[5], had MMSE ≥ 24 and CDR = 0.5, and showed memory impairment as assessed via age-sex-education-adjusted scores on at least one of the following tests: Rey's word list immediate and delayed recall[6] and Prose memory, immediate and delayed[7]. Participants with SMD stated that their memory function has deteriorated compared to earlier stages in life, reported that the time of onset was in adulthood, had a score of 25 or more on the Memory Complaint Questionnaire (MAC-Q)[8], and showed normal objective memory performance on Rey's and Prose memory tests[9]. Depression at screening was assessed with the Geriatric Depression Scale (GDS)[10], and participants with a GDS score of 6 or more were considered depressed and excluded from the study. The patients on treatment with cerebro-active drugs underwent a washout period of at least 14 days before assessment. Subjects were sampled including risk factors for AD, as well as different types of medication used to treat comorbidities in AD. To summarize, MCI subjects showed both subjective and objective memory impairment, SMD participants presented only memory complaints with a normal

score on the memory tests, and CN showed neither subjective nor objective memory impairment. To rule out other potential causes of cognitive impairment, all participants underwent blood tests (including full blood count, erythrocyte sedimentation rate, urea and electrolytes, thyroid function, vitamin B12, and folate); furthermore, all patients with AD and MCI, and 28 out of 40 subjects with SMD underwent magnetic resonance imaging (MRI) or computed tomography (CT) when MRI was contraindicated. Nine patients with diagnosis of possible AD underwent amyloid PET to confirm the diagnosis of probable AD with evidence of the AD pathophysiological process.

Exclusion criteria were as follows: significant chronic medical condition interfering with cognitive performances; visual and auditory acuity inadequate for neuropsychological testing; Geriatric Depression Scale greater than 6; alcohol intake of more than 4 units/day; absence of written informed consent from participant or caregiver; undocumented cognitive decline in patient with possible AD.

Of 250 enrolled subjects, 50 subjects were excluded according to above exclusion criteria, and 29 sera gave NMR spectra unsuitable for analysis, amounting to a total of 171 subjects considered in the study (Figure 1, main text). The final 171 participants were divided into four groups: 40 with Alzheimer's clinical syndrome (AD), 40 with amnestic MCI, 40 with SMC and 51 CN subjects (see Tables 1 and 2, main text for details). The effects of comorbidities and pharmacological treatments were not statistically significant (Tables 1 and 2, main text). Multivariate modeling PCA, applied to verify homogeneity of each sample group and the presence of possible outliers, placed all samples in the 95% confidence ellipse in the PCA graphical representation with no outliers (Fig. S1 and Table S1).

NMR spectroscopy measurements

All spectra were recorded on a 600-MHz Bruker Avance-III spectrometer (Bruker BioSpin GmbH, Rheinstetten, Germany) equipped with a CryoProbe. Two 1D proton spectra were acquired for each serum sample at a probe temperature of 300 K (27°C): 1) spectrum; and 2) a T_2-edited spectrum where signals from proteins and others macromolecules were attenuated with use of short spin-spin relaxation times employing the Carr-Purcell-Meiboom-Gill (CPMG) pulse sequence with water presaturation[11], and using a fixed inter echo delay to eliminate diffusion and J-modulation effects. These spectra were used for multivariate analysis. 1D ^{1}H-NMR spectra were collected at 300 K with the excitation sculpting pulse sequence to suppress the water resonance[12]. We used a double-pulsed field gradient echo, with a soft square pulse of 4 ms at the water resonance frequency and gradient pulses of 1 ms each in duration, adding 128 transients of 64k complex points, with an acquisition time of 4 s/transient. Time-domain data were all zero-filled to 128k complex points, and before Fourier transformation, an exponential multiplication of 0.6 Hz was applied. 2D clean TOCSY spectra were recorded by using a standard pulse sequence[13], and incorporating the excitation sculpting sequence for water suppression. In general, 320 equally spaced evolution time period t_1 values were acquired, averaging 4 transients of 2048 points. Time-domain data matrices were all zero-filled to 4096 points in both dimensions, and before Fourier transformation, a Lorentz-to-Gauss window with different parameters was applied for both t_1 and t_2 dimensions for all the experiments. The spectral positions of the lines ("resonances") in both homonuclear 1D and 2D spectra were referenced to the spectral position of the signal originating from 0.10 mmol/L TSP, which was assumed to resonate at a δ value of 0.00 ppm.

For the natural abundance of 2D ^{1}H-^{13}C HSQC spectra, we used an echo-antiecho phase sensitive pulse sequence by using adiabatic pulses for decoupling[14]. One hundred twenty-

eight equally spaced evolution–time period t_1 values were acquired, averaging 48 transients of 2048 points and using GARP4 for decoupling. The final data matrix was zero-filled to 4096 in both dimensions and apodized before Fourier transformation by a shifted cosine window function in t_2 and in t_1. Linear prediction was also applied to extend the data to twice their length in t_1. The spectral positions of the ‘‘resonances’’ were referenced to the lactate signal (βCH_3), which was assumed to resonate at 1.33 ppm for ^1H and 20.76 ppm for ^{13}C.

REFERENCES

1. Guigoz, Y., B. Vellas, and J.P. Garry. 1994. Mini Nutritional Assessment: A practical assessment tool for grading the nutritional state of elderly patients. *Facts Res. Gerontol.* Suppl. 2:15–59.

2. McKhann, G.M., D.S. Knopman, H. Chertkow, B.T. Hyman, C.R. Jr Jack, C.H. Kawas, W.E. Klunk, W.J. Koroshetz, J.J. Manly, R. Mayeux, et al. 2011. The diagnosis of dementia due to Alzheimer's disease: recommendations from the National Institute on Aging-Alzheimer's Association workgroups on diagnostic guidelines for Alzheimer's disease. *Alzheimers Dement.* 7:263–269. https://doi.org/10.1016/j.jalz.2011.03.005

3. Folstein, M.F., S.E. Folstein, and P.R. McHugh. 1975. "Minimental state": a practical method for grading the cognitive state of patients for the clinician. *J. Psychiatr. Res.* 12:189–198.

4. Berg, L. 1988. Clinical Dementia Rating (CDR). *Psychopharmacol. Bull.* 24:637–639.

5. Albert M.S., S.T. DeKosky, D. Dickson, B. Dubois, H.H. Feldman, N.C. Fox, A. Gamst, D.M. Holtzman, W.J. Jagust, R.C. Petersen, et al. 2011. The diagnosis of mild cognitive impairment due to Alzheimer's disease: recommendations from the National Institute on Aging-Alzheimer's Association workgroups on diagnostic guidelines for Alzheimer's disease. *Alzheimers Dement.* 7:270–279. https://doi.org/10.1016/j.jalz.2011.03.008

6. Rey, A. 1958. L'examen clinique en psychologie: Memorisation d'une serie de 15 mots en 5 repetitions. Paris: Universitaires des France Presses.

7. Spinnler, H. and G. Tognoni. 1987. Standardizzazione e taratura italiana di test neuropsicologici. *Ital. J. Neurol. Sci.* 8:1–120.

8. Crook, T.H. 3rd, E.P. Feher, and G.J. Larrabee. 1992. Assessment of memory complaint in age-associated memory impairment: the MAC-Q. *Int. Psychogeriatr.* 4:165–176.

9. Abdulrab, K., and R. Heun. 2008. Subjective Memory Impairment. A review of its definitions indicates the need for a comprehensive set of standardised and validated criteria. *Eur. Psychiatry* 23:321–330. https://doi.org/10.1016/j.eurpsy.2008.02.004

10. Almeida, O.P., and S.A. Almeida. 1999. Short versions of the geriatric depression scale: a study of their validity for the diagnosis of a major depressive episode according to ICD-10 and DSM-IV. *Int. J. Geriatr. Psychiatry* 14:858–865.

11. de Graaf, R.A., and K.L. Beha. 2003. Quantitative ^{1}H NMR spectroscopy of blood plasma metabolites. *Anal. Chem.* 75:2100–2104. https://doi.org/10.1021/ac020782+

12. Hwang, T.-L., and A.J. Shaka. 1995. Water suppression that works: excitation sculpting using arbitrary waveforms and pulse field gradients. *J. Magn. Reson.* 112:275–279. https://doi.org/10.1006/jmra.1995.1047
13. Griesinger, C., G. Otting, K. Wüthrich, and R.R. Ernst. 1988. Clean TOCSY for proton spin system identification in macromolecules. *J. Am. Chem. Soc.* 110:7870–7872. https://doi.org/10.1021/ja00231a044
14. Kay, L.E., P. Keifer, and T. Saarinen. 1992. Pure absorption gradient enhanced heteronuclear single quantum correlation spectroscopy with improved sensitivity. *J. Am. Chem. Soc.* 114:10663–10665. https://doi.org/10.1021/ja00052a088

SUPPLEMENTARY FIGURES

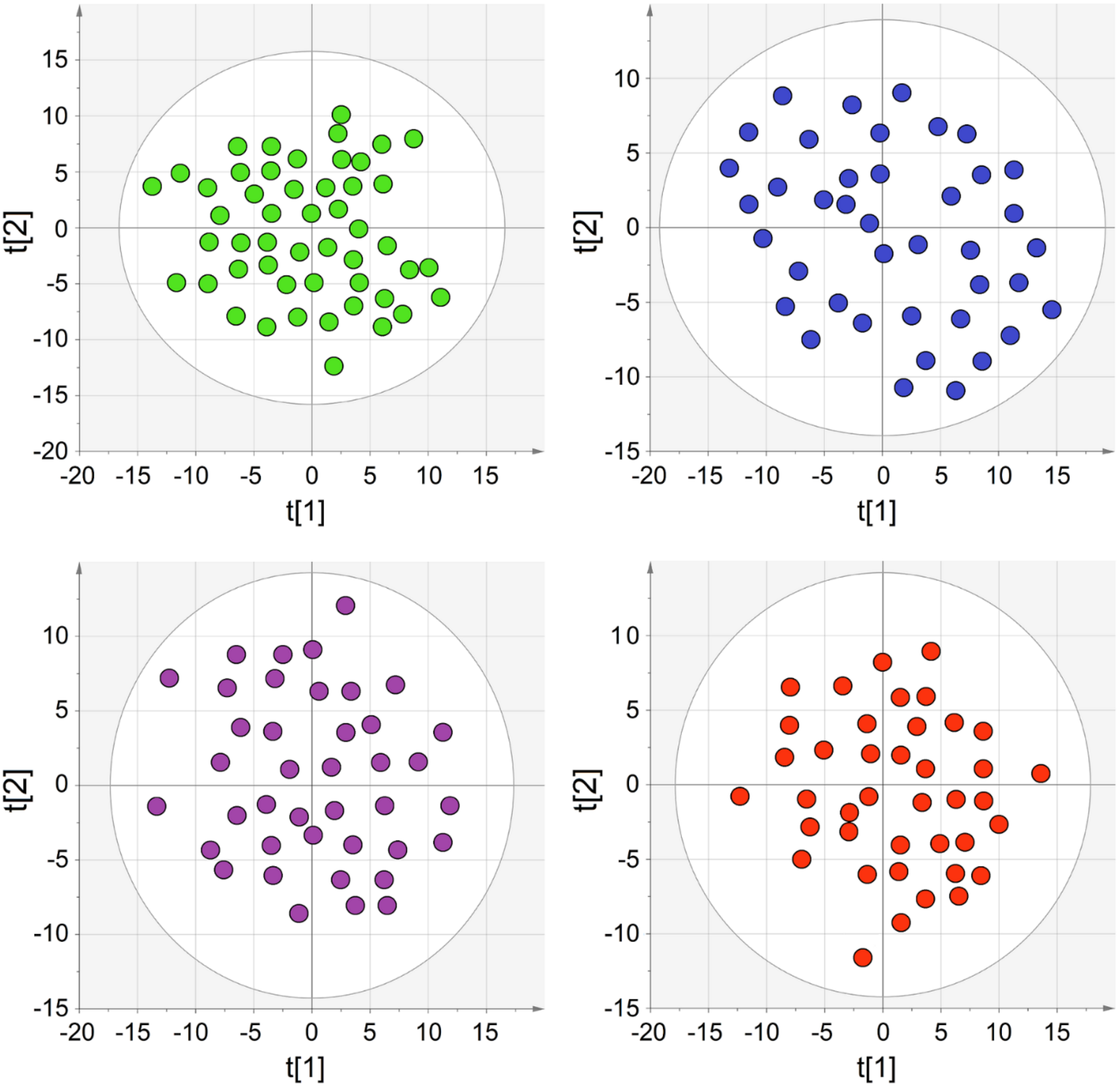

Fig. S1. *PCA scores plots representing each single class model for all sample sets (90 training and 81 validation samples). CN, cognitively normal subjects (green dots); SMD, subjective memory decline (blue dots); MCI, mild cognitive impairment (purple dots); and AD, Alzheimer's disease (red dots). For each class-model, no outliers were detected, confirming classes' homogeneity. The labels t[1] and t[2] along the axes represent the scores (the first 2 partial least-squares components) of the model, which are sufficient to build a satisfactory classification model.*

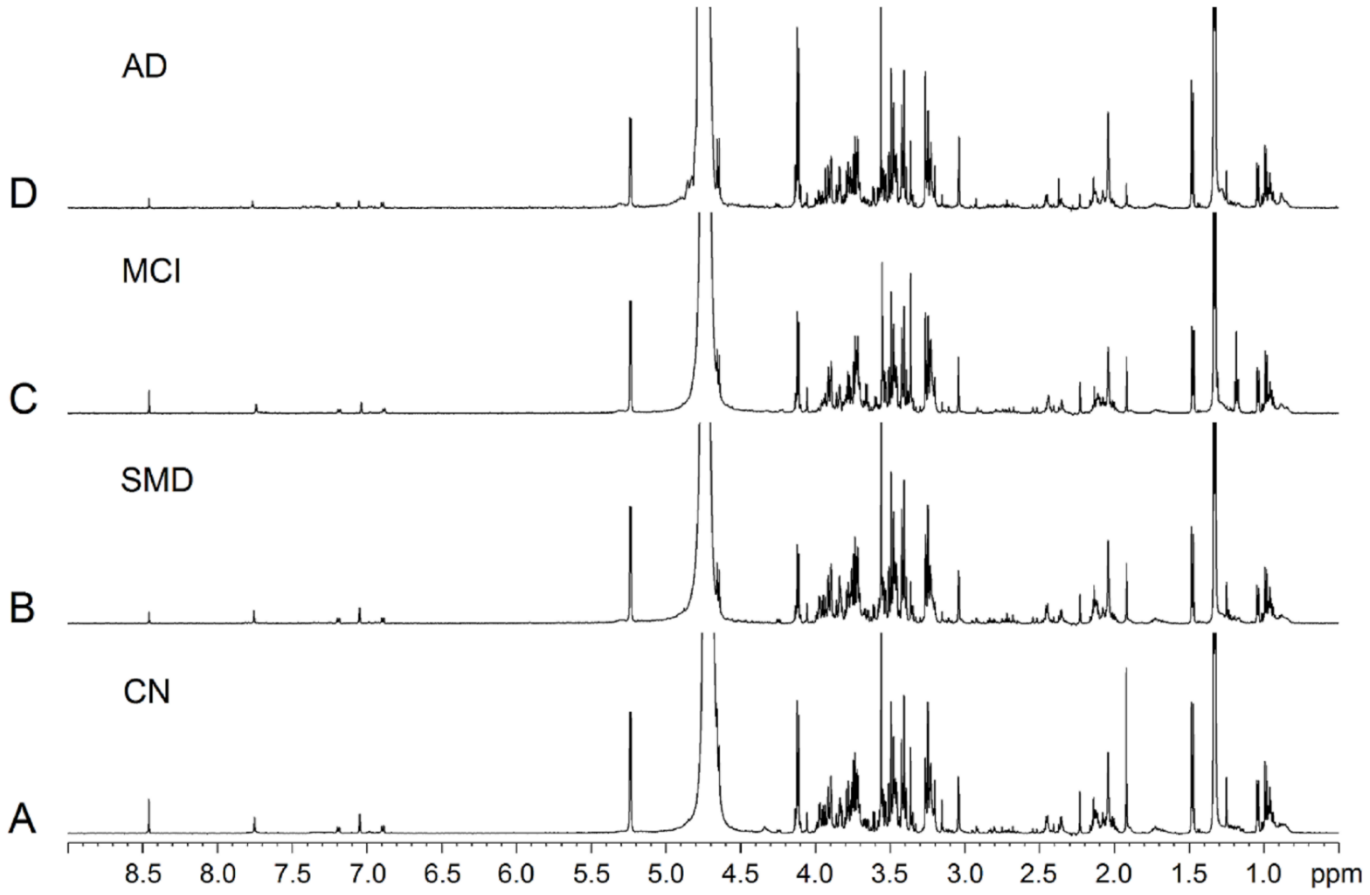

Fig. S2. *NMR spectra of serum samples. Representative 1D ^{1}H spectra of a CN patient (A), SMD subject (B), MCI subject (C), and AD patient (D). Signals were assigned to single metabolites (Table S2) by resorting to 2D NMR experiments and referring to published data on metabolite chemical shifts. Intensity is plotted on the y-axis, and magnetic field strength is plotted on the x-axis that usually ranges from 0 to 12 ppm.*

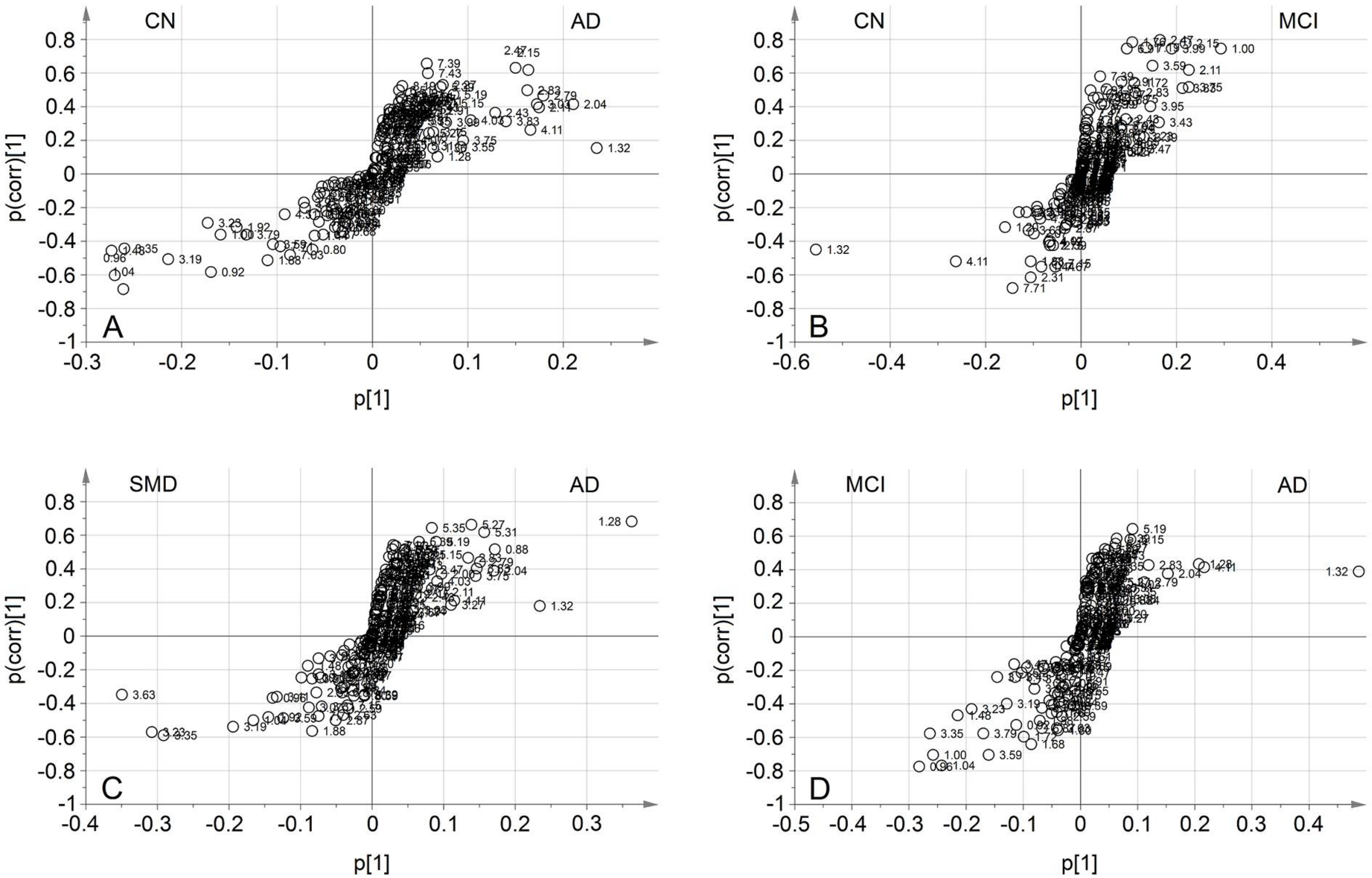

Fig. S3. *S-plots reporting* p_{corr} *against the predictive loading vector p of the model: A, CN vs. AD; B, CN vs. MCI; C, SMD vs. AD; D, MCI vs. AD. Numbers refer to chemical shift values (i.e., spectral position) of the metabolites reported in Fig. 2 (see main text).*

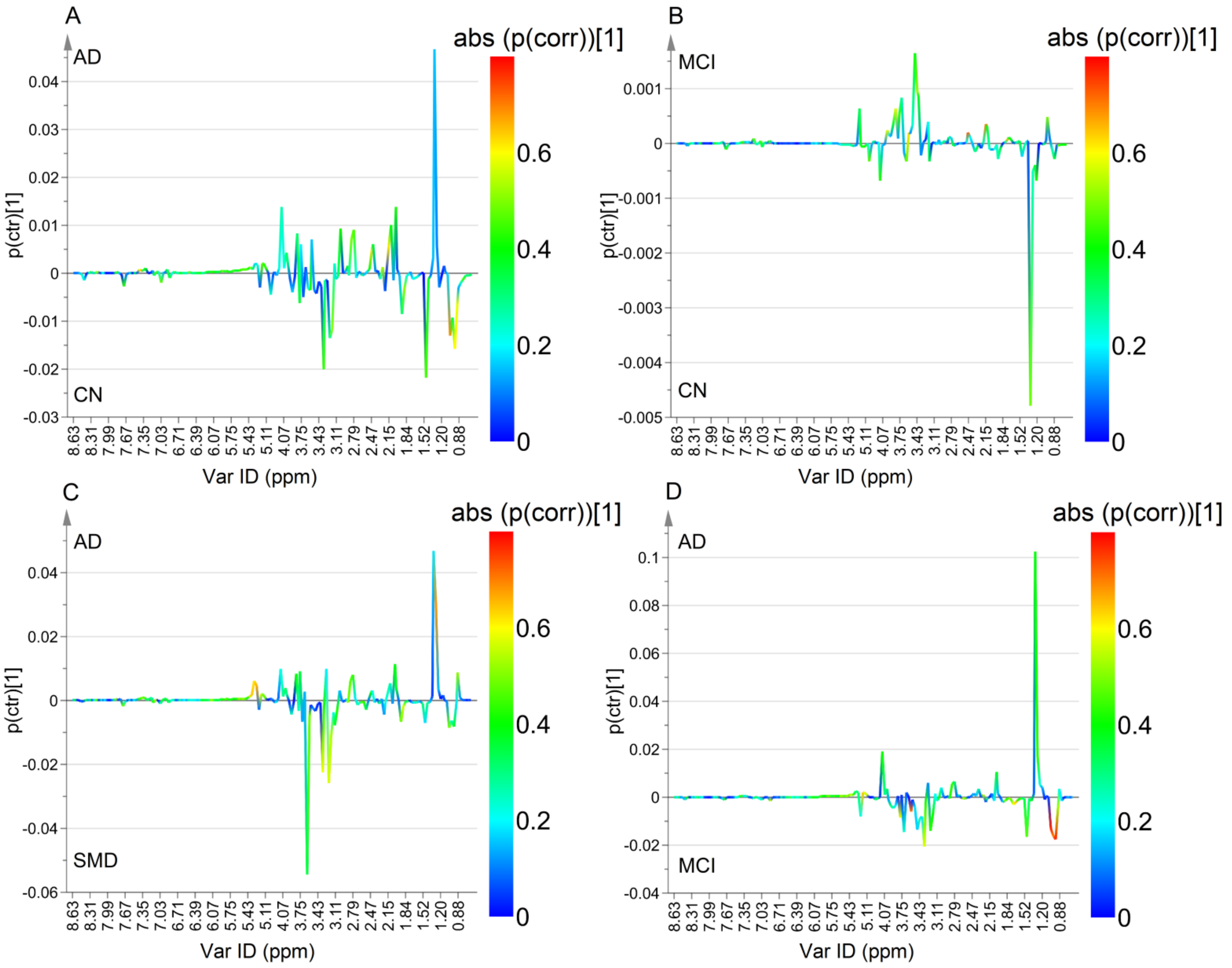

Fig. S4. *S-line plots between 8.7 and 0.5 ppm corresponding to the Fig S3A-D models. Positive lines represent metabolites that increase their concentration in the indicated classes, while negative lines define diminished concentrations. The buckets are reported on the x-axis (Var ID, in ppm; refer to Table S2 for assignments); the y-axis p(ctr)[1] refers to the loading value for each variable according to the centering, while abs (p[corr])[1] indicates the absolute correlation value, which is reflected by the peack colors.*

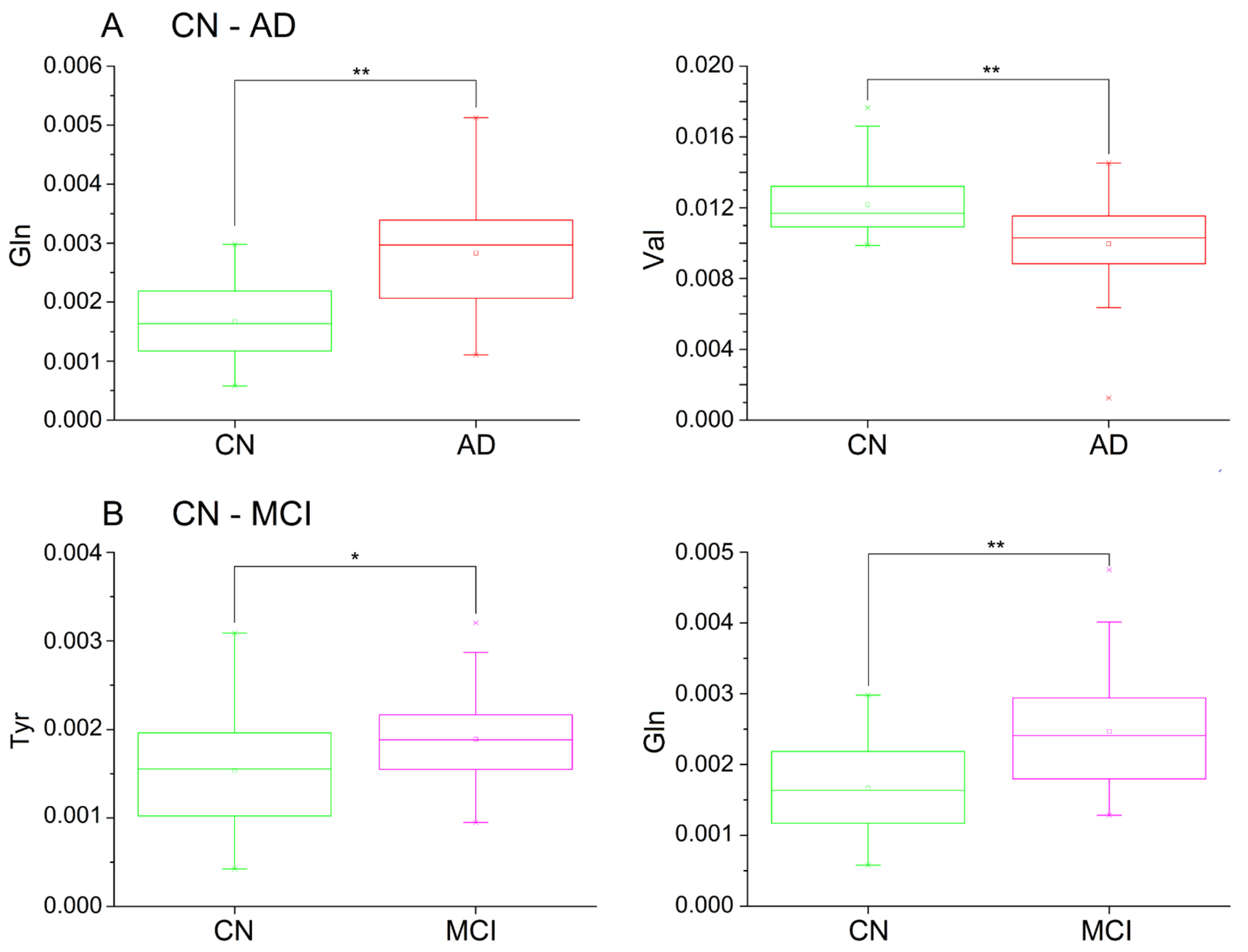

Fig. S5. *Box-and-whisker plots showing the concentration levels of the metabolites presented in the ROC curves of Figures 4A and 4B. A, glutamine (Gln) and valine (Val) for the CN-AD model; B, tyrosine (Tyr) and glutamine (Gln) for the CN-MCI model. Boxes show median (horizontal line in each box), the mean (the empty box), 25th and 75th percentiles (edges of box), maximum and minimum values (whiskers), and the outliers (cross). ANOVA test significance is reported as *, $p < 0.05$ and **, $p < 0.001$.*

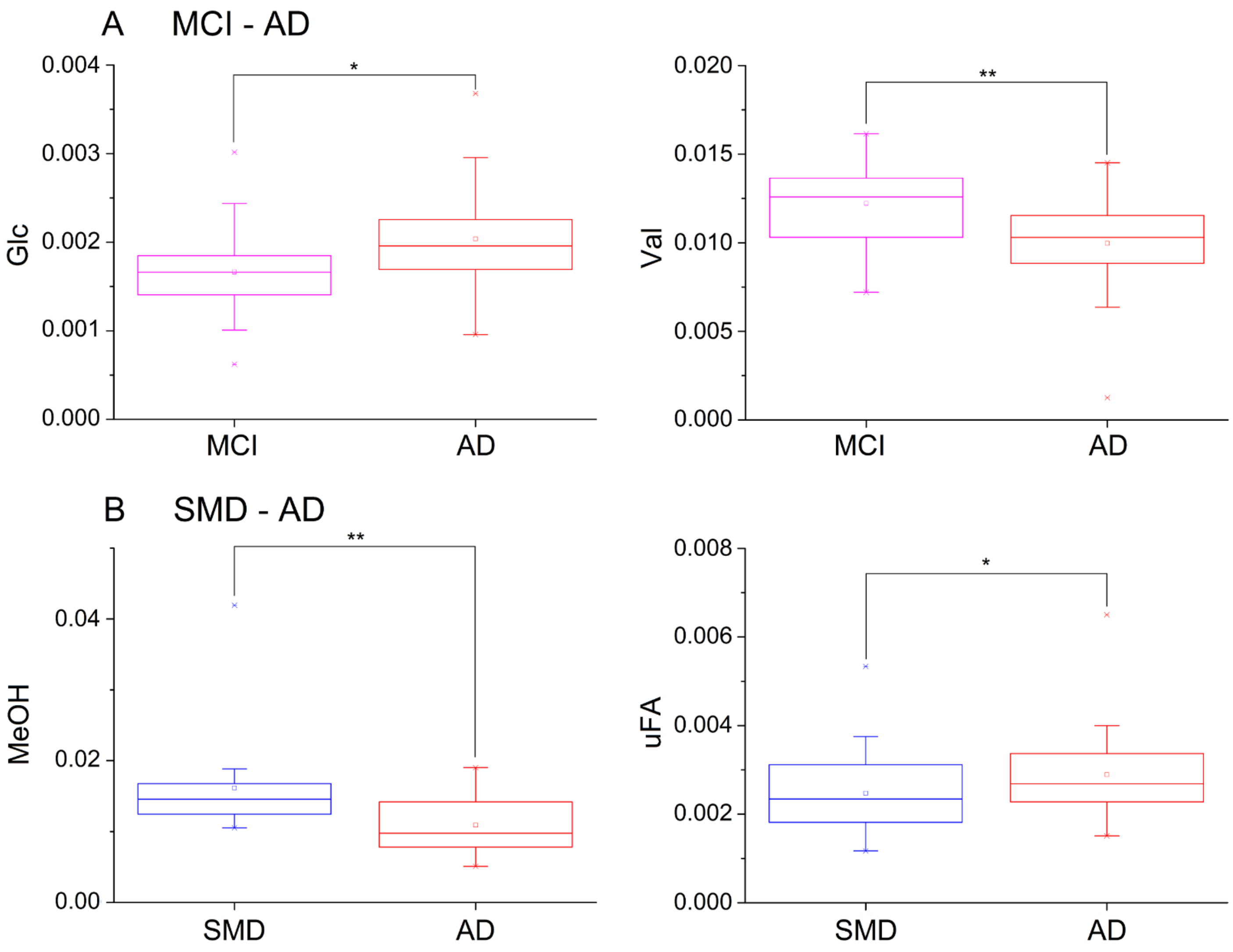

Fig. S6. *Box-and-whisker plots showing the concentration levels of the metabolites presented in the ROC curves of Figures 4C and 4D (see main text). A, glucose (Glc) and valine (Val) for the MCI-AD model; B, methanol (MeOH) and unsaturated fatty acids (uFA) for the SMD-AD model. ANOVA test significance is reported as *, $p < 0.05$ and **, $p < 0.001$.*

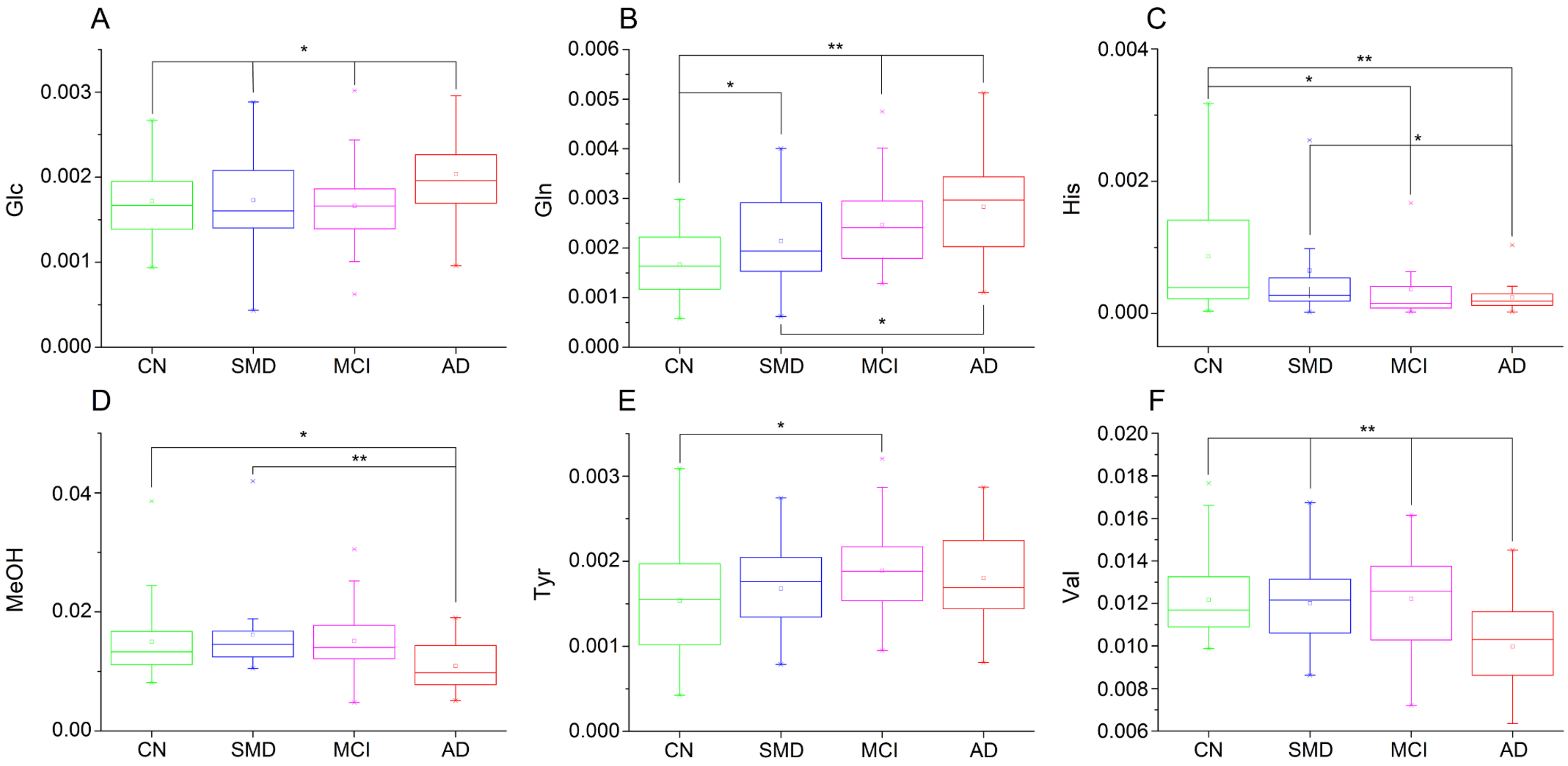

Fig. S7. *Box-and-whisker plots showing the concentration levels of selected metabolites evaluated for CN, SMD, MCI, and AD sample classes. A, glucose (Glc); B, glutamine (Gln); C, histidine (His); D, methanol (MeOH); E, tyrosine (Tyr); and F, valine (Val). ANOVA Test significance is reported as *, $p < 0.05$ and **, $p < 0.001$.*

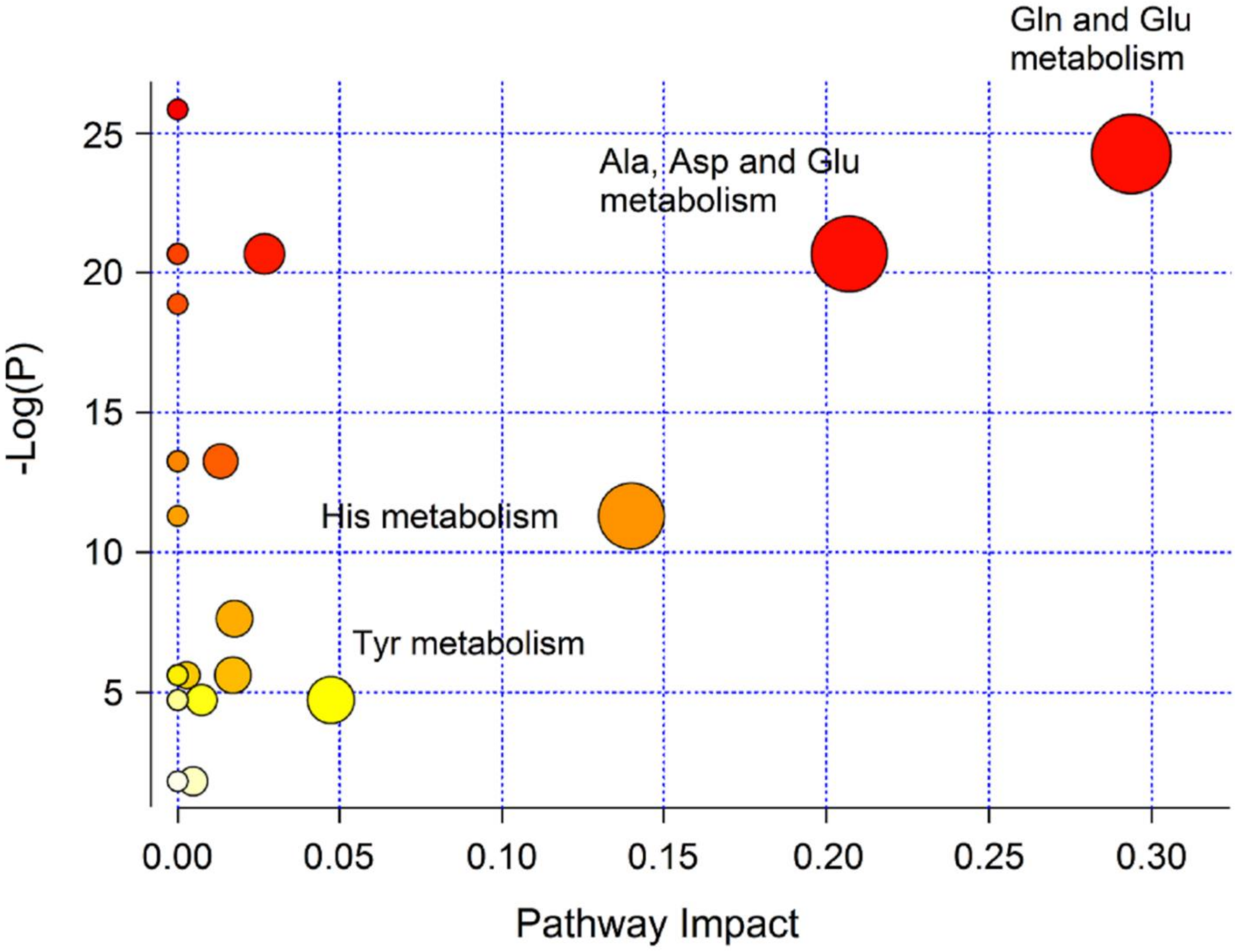

Fig. S8. *Pathway analysis overview showing the altered metabolic pathways associated with all blood markers derived from discriminant class analysis involving CN, SMD, MCI and AD, and presenting both VIP ≥ 1 and |p_{corr}| ≥ 0.6. The most relevant networks are indicated according to the pathway impact and the p value.*

SUPPLEMENTARY TABLES

Table S1. PCA model parameters for each class of samples of the training set[a]

PCA Class	*N subjects*	*Model Components*	*Model Parameters*
CN	28	2	R^2 = 0.410; Q^2 = 0.240
SMD	20	2	R^2 = 0.410; Q^2 = 0.174
MCI	21	2	R^2 = 0.372; Q^2 = 0.122
AD	21	2	R^2 = 0.384; Q^2 = 0.184

[a]PCA, principal component analysis; CN, cognitively normal; SMD, subjective memory decline; MCI, mild cognitive impairment; AD, Alzheimer's disease; N = number of samples; R^2 = the goodness-of-fit parameter, measure of how well the model fits the data; Q^2 = goodness-of-prediction parameter, measure of how well the model predicts new data. For R^2 and Q^2 acceptable values must be ≥ 0.5, with $|R^2 - Q^2| < 0.2–0.3$.

Table S2. Proton NMR assignments of the compounds identified in human serum samples[a]

N	*Metabolites*	*^{1}H chemical shift (ppm)(multiplicity)*
1	HDL	0.84(t)
2	VLDL	0.88(t)
3	2-Hydroxybutyrate	0.89(t), 1.64(m), 1.71(m), 3.99(m)
4	Isoleucine	0.92(t), 0.96(d), 1.24(m), 1.45(m), 1.93(m), 3.62(d)
5	Leucine	0.93(d), 1.00(d), 1.63(m), 1.70(m)
6	Valine	0.96(d), 1.04(d), 2.21(m)
7	Isobutyrate	1.05(d), 2.37(m)
8	Isopropanol	1.17(m), 3.97(d)
9	Ethanol	1.18(t), 3.65(q)
10	3-Hydroxybutytrate	1.16(d), 2.33(m), 2.40(m), 4.14(m)
11	Methylmalonate	1.22(d), 3.13(q)
12	3-Hidroxyisovalerate	1.24(s), 2.35(s)
13	Lipid	1.25(m), 1.57(m), 2.01(m), 2.24(m), 2.74(m)
14	Threonine	1.31(d), 3.58(d), 4.26(m)
15	Lactate	1.33(d), 4.11(q)
16	Alanine	1.48(d), 3.76(q)
17	Arginine	1.64(m), 1.71(m), 1.83(m), 1.86(m), 3.23(t), 3.66(t)
18	Lysine	1.40(m), 1.44(m), 1.72(m), 1.88(m), 3.04(t), 3.69(m)
19	Acetate	1.91(s)
20	Proline	1.95(m), 2.02(m), 2.06(m), 2.30(m), 3.29(m), 4.15(m)
21	Glutamate	2.08(m), 2.32(m), 3.75(t)
22	Glutamine	2.15(m), 2.47(m), 3.77(t)
23	Methionine	2.13(s), 2.24(m),2.64(t), 3.85(t)
24	Glycoprotein	2.18(s)
25	Acetone	2.23(s)
26	Acetoacetate	2.29(s), 3.41(s)
27	Pyruvate	2.38(s)
28	Succinate	2.40(s)
29	Carnitine	2.41(dd), 3.43(s), 2.44(dd), 3.40(m), 4.57(m)
30	Citrate	2.52(dd), 2.68(dd)
31	Aspartate	2.64(dd), 2.78(dd), 3.89(t)
32	Sarcosine	2.69(s), 3.59(s)
33	Methylguanidine	2.81(s), 3.35(s)
34	Asparagine	2.87(dd), 2.95(dd), 4.02(dd)
35	Trimethylamine	2.90(s)
36	Dimethylglycine	2.91(s), 3.71(s)
37	Tyramine	2.93(t), 3.23(t), 6.90(d), 7.20(d)
38	Tyrosine	2.98(q), 6.87(d), 7.19(d),
39	Phosphocreatine	3.02(s), 3.94(s)
40	Creatine	3.03(s), 3.94(s)
41	Creatinine	3.04(s), 4.05(s)
42	Phenylalanine	3.15(dd), 3.23(dd), 3.98(dd), 7.28(d), 7.33(t), 7.41(t)
43	Choline	3.15(s), 3.44(dd), 3.99(m)
44	Histidine	3.14(dd), 3.25(dd), 4.03(dd), 6.99(s), 7.71(s)
45	1-Methylhistidine	3.20(m), 3.68(s), 3.96(dd), 7.03(s), 7.83(s)
46	O-Acetylcholine	3.21(s)
47	Taurine	3.23(t), 3.41(t)
48	O-Phosphocholine	3.24(s)
49	β-Glucose	3.25(dd), 3.40(t), 3.46(m), 3.71(m), 3.89(d), 4.64(d)
50	Trimetylamine N-oxide	3.26(s), 3.87(s)
51	Myo-inositol	3.27(t), 3.53(dd), 3.62(t), 4.05(t)
52	Tryptophan	3.30 (dd), 3.45(dd), 4.03(dd), 7.20(t), 7.28(t), 7.31(s), 7.53(d), 7.73(d)
53	Methanol	3.35(s)
54	Scyllo-inositol	3.37(s)

55	α-Glucose	3.40(m), 3.52(d), 3.74(t), 3.82(m), 5.19(d)
56	Glycine	3.49(s)
57	Serine	3.81(dd), 3.92(dd), 3.97(dd)
58	Triglycerides	4.06(m), 4.27(m), 5.20(m)
59	Unsaturated fatty acids	5.27(m)
60	Deoxycytidine triphosphate	6.07(d), 6.08(t), 7.83(d)
61	Hypoxanthine	8.09(s), 8.19(s)
62	Formate	8.45(s)

[a]HDL, high density lipoprotein; VLDL, very low density lipoprotein, s, singlet; d, doublet; dd, double doublet; t, triplet; q, quartet; m, multiplet.

Table S3. Modeling diagnostics of the statistical significant OPLS-DA models

Training set model validation					
OPLS-DA model	***N subjects***	***Predictive (p) and orthogonal (o) components***	***Internal validation***[a]	***Response permutation (800 hits)***[b]	***CV-ANOVA p value***[c]
Model 1: CN–AD	28–21	1p + 2o	$R^2X = 0.457$; $R^2Y = 0.747$; $Q^2c = 0.428$	$R^2 = 0.422$; $Q^2 = -0.522$	0.0004
Model 2: CN–MCI	28–21	1p + 1o	$R^2X = 0.424$; $R^2Y = 0.422$; $Q^2c = 0.053$	$R^2 = 0.314$; $Q^2 = -0.298$	0.039
Model 3: SMD–AD	20–21	1p + 1o	$R^2X = 0.346$; $R^2Y = 0.617$; $Q^2c = 0.355$	$R^2 = 0.361$; $Q^2 = -0.362$	0.002
Model 4: MCI–AD	21–21	1p + 1o	$R^2X = 0.443$; $R^2Y = 0.594$; $Q^2c = 0.178$	$R^2 = 0.333$; $Q^2 = -0.358$	0.01
All-class model: CN–SMD–MCI–AD	90	3p + 1o	$R^2X = 0.383$; $R^2Y = 0.376$; $Q^2c = 0.094$	$R^2 = 0.268$; $Q^2 = -0.243$	0.90

[a]R^2X, fraction of NMR data variation modeled; R^2, measure of how good is class-separation modeled; Q^2c, cross-validated R^2.
[b]R^2 and Q^2, intercept values obtained from the permuation test.
[c]*p* value for the OPLS-DA reliability ($p < 0.05$).

Table S4. Variable selection from each OPLS-DA statistical model for single and multiple ROC curve analysis[a]

Variables selection for ROC curves											
Model 1: CN-AD						***Model 2: CN-MCI***					
**CN**			**AD**			**CN**			**MCI**		
**Signal**	**Var (ppm)**	p_{corr}	**Signal**	**Var (ppm)**	p_{corr}	**Signal**	**Var (ppm)**	p_{corr}	**Signal**	**Var (ppm)**	p_{corr}
**Val**	1.04	-0.69	**Gln**	2.47	0.63	**His**	7.71	-0.68	**Tyr**	7.19	0.75
Leu/Val	0.96	-0.61	**Gln**	2.15	0.62	Glu	2.32	-0.61	Ile/Leu	1.00	0.74
Ile/Leu	0.92	-0.62							**Gln**	2.47	0.63
Model 3: SMD-AD						***Model 4: MCI-AD***					
**SMD**			**AD**			**MCI**			**AD**		
**Signal**	**Var (ppm)**	p_{corr}	**Signal**	**Var (ppm)**	p_{corr}	**Signal**	**Var (ppm)**	p_{corr}	**Signal**	**Var (ppm)**	p_{corr}
**MeOH**	3.35	-0.61	**uFA**	5.27	0.66	**Val**	1.04	-0.77	**Glc**	5.19	0.64
						Leu/Val	0.96	-0.77			
						Ile/Leu	1.00	-0.70			

[a]Signals with VIP > 1, $|p_{corr}| \geq \pm 0.6$ and resonating in isolated buckets (metabolites in bold) were chosen as representative markers for sample class characterization: valine (Val, 1.04 ppm), glutamine (Gln, 2.47 and 2.15 ppm), histidine (His, 7.71 ppm), tyrosine (Tyr, 7.19 ppm); glucose (Glc, 5.19 ppm), methanol (MeOH, 3.35 ppm) and unsaturated fatty acids (uFA, 5.27 ppm). Although all variables have VIP > 1 and $|p_{corr}| \geq \pm 0.6$, only those in bold were considered for ROC analysis since for the others the corresponding buckets presented partial peak overlap thus hampering a reliable quantification.
